# Bleaching Susceptibility and Resistance of Octocorals and Anemones at the World’s Southern-Most Coral Reef

**DOI:** 10.3389/fphys.2022.804193

**Published:** 2022-05-19

**Authors:** Rosemary K Steinberg, Tracy D Ainsworth, Tess Moriarty, Teresa Bednarek, Katherine A Dafforn, Emma L Johnston

**Affiliations:** ^1^ Evolution and Ecology Research Centre and Centre for Marine Science and Innovation, School of Biological, Earth, and Environmental Sciences, Faculty of Science, University of New South Wales, Sydney, NSW, Australia; ^2^ Sydney Institute of Marine Science, Mosman, NSW, Australia; ^3^ School of Environmental and Life Sciences, The University of Newcastle, Ourimbah, NSW, Australia; ^4^ RUHR Universtad Bouchum, Bouchum, Germany; ^5^ School of Natural Sciences, Macquarie University, Sydney, NSW, Australia

**Keywords:** soft coral, alcyonacea, chlorophyll, coral bleaching, symbiodiniaceae, marine heatwave

## Abstract

Coral reefs are amongst the most biodiverse ecosystems on earth, and while stony corals create the foundational complexity of these ecosystems, octocorals and anemones contribute significantly to their biodiversity and function. Like stony corals, many octocorals contain Symbiodiniaceae endosymbionts and can bleach when temperatures exceed the species’ upper thermal limit. Here, we report octocoral bleaching susceptibility and resistance within the subtropical Lord Howe Island coral reef ecosystem during and after marine heatwaves in 2019. Octocoral and anemone surveys were conducted at multiple reef locations within the Lord Howe Island lagoon during, immediately after, and 7 months after the heatwaves. One octocoral species, *Cladiella* sp. 1, experienced bleaching and mortality, with some bleached colonies detaching from the reef structure during the heatwave (presumed dead). Those that remained attached to the benthos survived the event and recovered endosymbionts within 7 months of bleaching. *Cladiella* sp. 1 Symbiodiniaceae density (in cells per µg protein), chlorophyll *a* and *c*_*2*_ per µg protein, and photosynthetic efficiency were significantly lower in bleached colonies compared to unbleached colonies, while chlorophyll *a* and *c*_*2*_ per symbiont were higher. Interestingly, no other symbiotic octocoral species of the Lord Howe Island lagoonal reef bleached. Unbleached *Xenia* cf *crassa* colonies had higher Symbiodiniaceae and chlorophyll densities during the marine heatwave compared to other monitoring intervals, while *Cladiella* sp. 2 densities did not change substantially through time. Previous work on octocoral bleaching has focused primarily on gorgonian octocorals, while this study provides insight into bleaching variability in other octocoral groups. The study also provides further evidence that octocorals may be generally more resistant to bleaching than stony corals in many, but not all, reef ecosystems. Responses to marine heating events vary and should be assessed on a species by species basis.

## 1 Introduction

Tropical and subtropical coral reefs are under increasing threat due to human induced climate change as they bleach in response to a variety of stressors, notably thermal stress (Harrison et al., 2011; Hughes et al., 2017; Suggett and Smith, 2020). Coral bleaching is the breakdown of the partnership between endosymbiotic dinoflagellates of the family Symbiodiniaceae and their cnidarian coral host. Bleaching is often measured as a loss in endosymbiont density within the coral or loss of chlorophyll from the Symbiodiniaceae themselves (Coles and Jokiel, 1978; Kleppel et al., 1989; Gates et al., 1992; Brown, 1997; Hoegh-Guldberg, 1999). The consequences of coral bleaching on coral reefs include extensive coral mortality, reduced fecundity and recruitment rates, and loss of associated function and biodiversity (Michalek-Wagner and Willis, 2001a; Loya et al., 2001; Pratchett et al., 2008; Stuart-Smith et al., 2018; Hughes et al., 2019; Donovan et al., 2021; González-barrios and Cabral-tena, 2021). The consequences of bleaching can be affected by local stressors, with turbidity, wave exposure, macroalgae cover, and urchin abundance all significantly affecting changes in coral cover in the year after heat-induced bleaching (Donovan et al., 2021). The frequency of coral bleaching has increased in recent years, with annual bleaching events predicted by 2055 and periods of annual bleaching already occurring on some reefs (Van Hooidonk et al., 2014; Hughes et al., 2017; Slattery et al., 2019). Bleaching susceptibility differs between cnidarian groups (anemone, octocoral, stony coral, etc.) and between stony coral species, where growth forms and even size classes within species are reported to have differential bleaching susceptibilities (Loya et al., 2001; Brandt, 2009; Fabricius et al., 2011; Grottoli et al., 2014).

While stony corals are generally the focus of bleaching studies across coral reef ecosystems, other cnidarians supporting photoendosymbionts, such as octocorals and anemones, are also susceptible (Loya et al., 2001; Saenz-Agudelo et al., 2011; Hill et al., 2014; Maucieri and Baum, 2021). In fact, while some species of massive stony corals, such as Poritid corals, are sometimes considered “winners” under increased sea surface temperatures, branching stony corals and octocorals have previously been considered “losers” during bleaching events (Loya et al., 2001). The consequences of coral bleaching on individual octocoral colonies include loss of photoendosymbionts and chlorophyll, changes in Symbiodiniaceae community structure, increase in production of nitric oxide and heat shock proteins by Symbiodiniaceae, changes to lipid, protein, and metabolite content and composition, and more (Michalek-Wagner and Willis, 2001a; Ross, 2014; Panithanarak, 2015; Farag et al., 2018; Sikorskaya et al., 2020; Maucieri and Baum, 2021). Thus, to truly understand the impacts of climate change on coral reef systems and individual colonies, it is necessary to include a range of cnidarians in bleaching studies.

Although octocorals and anemones are not typically considered “reef builders”, they do share a role as soft benthic habitat formers. Both are important members of benthic communities in reef and non-reef environments, including artificial habitats (Perkol-Finkel and Benayahu, 2004; Baillon et al., 2012; Ng et al., 2015; Epstein and Kingsford, 2019). Octocorals and anemones are attractive habitat for mobile vertebrate and invertebrate species, including generalists and obligate mutualists (Elliott and Mariscal, 2001; Lourie and Randall, 2003; Valdivia and Stotz, 2006; Huebner et al., 2012; Poulos et al., 2013). In fact, fish species diversity at two reefs in the GBR increased with increasing octocoral, but not stony coral, cover (Epstein and Kingsford, 2019). Both are also important food sources for many species and are especially popular with butterflyfishes (Pratchett, 2007; Slattery and Gochfeld, 2016; Epstein and Kingsford, 2019). However, many studies of coral reef community structure and bleaching do not consider octocorals (exceptions are reviewed in Steinberg et al. (2020) and below), leading to a critical knowledge gap of how they might respond to global stressors. Both stony corals and anemones have previously been recorded as bleaching at Lord Howe Island (Harrison et al., 2011; Boulotte et al., 2016); however, octocoral response to and recovery from thermal bleaching in subtropical reef systems in Australia, such as Lord Howe Island, are unknown.

Of the global studies that have considered octocorals, most report a link between ocean warming and bleaching and mass mortality and many describe variability by site and taxa (Loya et al., 2001; Goulet et al., 2008a; Prada et al., 2010; Dias and Gondim, 2016; Maucieri and Baum, 2021). In Japan, Loya et al. (2001) reported declines of the two dominant octocoral species on the reefs of Sesoko Island, Japan, and Maucieri and Baum. (2021) found that octocorals were lost completely from all surveyed sites at Kiritimati atoll during the 2015/2016 El Niño heatwave. In contrast, in the Caribbean octocorals are generally more resistant to bleaching than stony corals, but responses are variable between species and extreme heat can cause octocoral bleaching and mortality (Lasker, 2003, 2005; Drohan et al., 2005; Prada et al., 2010; Dias and Gondim, 2016; Goulet et al., 2017; Mccauley et al., 2018; Cerpovicz and Lasker, 2021). Octocorals on Puerto Rico and Brazil’s reefs varied greatly in bleaching susceptibility by genus (Prada et al., 2010; Dias and Gondim, 2016). On the reefs of the Paraíba coast, Brazil, a gorgonian octocoral bleached along with four stony species and one hydrocoral, but only the gorgonian exhibited mortality during the study period (Dias and Gondim, 2016). In reefs of southwest Puerto Rico, octocoral response to elevated water temperatures was also taxa dependent, and only one species experienced mortality (Prada et al., 2010). Heat-induced mortality of octocorals can also occur without prior visual signs of bleaching (Lasker, 2005). Overall, there is considerable variability in bleaching response across octocoral taxa, including mortality.

In the Caribbean, studies of octocoral bleaching have focused on gorgonian octocorals, while in other parts of the world studies focus on Alcyonaceans, which may affect geographic bleaching patterns. Additionally, octocorals show biogeographical patterns of Symbiodiniaceae clade distribution, which may affect bleaching susceptibility and resistance (Goulet et al., 2008b). On Australia’s Great Barrier Reef, extensive bleaching of stony coral species was recorded in 1998, 2004, 2016, 2017 and 2020, which has resulted in loss of stony coral cover across the ecosystem (Thompson and Dolman, 2010; Hughes et al., 2017, 2018a, 2018b; Stuart-Smith et al., 2018; Thiault et al., 2021). Octocorals were also affected, with 43% of inshore octocoral colonies bleached during the 1998 bleaching event and significant taxonomic variability observed (Goulet et al., 2008a). Similarly, bleaching events were recorded in the Austral summers of 2010/2011 and 2015/2016 for coral reef ecosystems of the Indian ocean along Australia’s west coast coral reefs, though the majority of studies did not quantify octocoral bleaching (Moore et al., 2012; Le Nohaïc et al., 2017). Octocoral bleaching was quantified at the isolated Scotts Reef of North-western Australia, where bleaching resulted in a decline of up to 80% in total coral cover, and 6 years after bleaching stony corals had begun recovering while octocorals cover had barely changed (Smith et al., 2008). As such, there is limited information currently available on the bleaching susceptibility for octocorals across the extent of Australia’s coral reef ecosystems.

Previously stony corals and anemones were reported to undergo bleaching within the Lord Howe Island coral reefs, but octocoral bleaching has not been recorded in this UNESCO world-heritage listed marine park (Harrison et al., 2011; Boulotte et al., 2016). A major difficulty when studying octocorals and anemones is that they often leave little or no traces of existence following a mortality event. The consequences of bleaching can therefore be difficult–if not impossible–to detect if the immediate impact of high sea surface temperature (SST) is not quantified and no prior population data exist. The lack of prior data is often the case for the understudied octocoral populations on coral reefs (Steinberg et al., 2020). The consequences of increased sea surface temperature to the coral reef ecosystem of Lord Howe Island may also extend beyond the immediate impacts of coral bleaching and coral mortality on reefs. Increased SST reduces the ability of coral colonies to heal from fragmentation or damage, increases susceptibility to infectious disease, has been linked to Symbiodiniaceae-coral relationship shifting to parasitism by the endosymbiotic dinoflagellate, and decreases fecundity and reproduction in the year following bleaching events (Michalek-Wagner and Willis, 2001a, 2001b; Ruiz-Moreno et al., 2012; Bonesso et al., 2017; Baker et al., 2018).

The reefs of the Lord Howe Island lagoon marine park are ecologically and socially important to the ecosystem and people of Lord Howe Island. The waters around Lord Howe Island rank fifth in the Indo-Pacific for endemism, with 7.2% endemic fish species, many of which are supported by the reef structure (Randall, 1998). In addition, the local marine environment is important for the 350 permanent residents of the island, with local businesses providing tourism accommodation, boating, fishing, snorkelling, and diving both in and out of the lagoon (Lord Howe Island Tourism Association, 2021). Residents and tourists are attracted to the unique and pristine natural environment and the island’s commitment to sustainability (Lord Howe Island Tourism Association, 2021), both of which may be negatively impacted by reef degradation. To better predict the impact of climate change and increasing sea surface temperatures within high latitude reefs it is important to understand how different species within the remote Lord Howe Island reef system are impacted by marine heatwaves. Here, we provide the first report of octocoral bleaching susceptibility and resilience in the world’s southernmost coral reef ecosystem, Lord Howe Island. In doing so, we provide insight into the impact of climate driven increasing SSTs and marine heatwave events to these remote, valuable, and understudied coral reef ecosystems.

## 2 Methods

### 2.1 Study Locations

**FIGURE 1:**
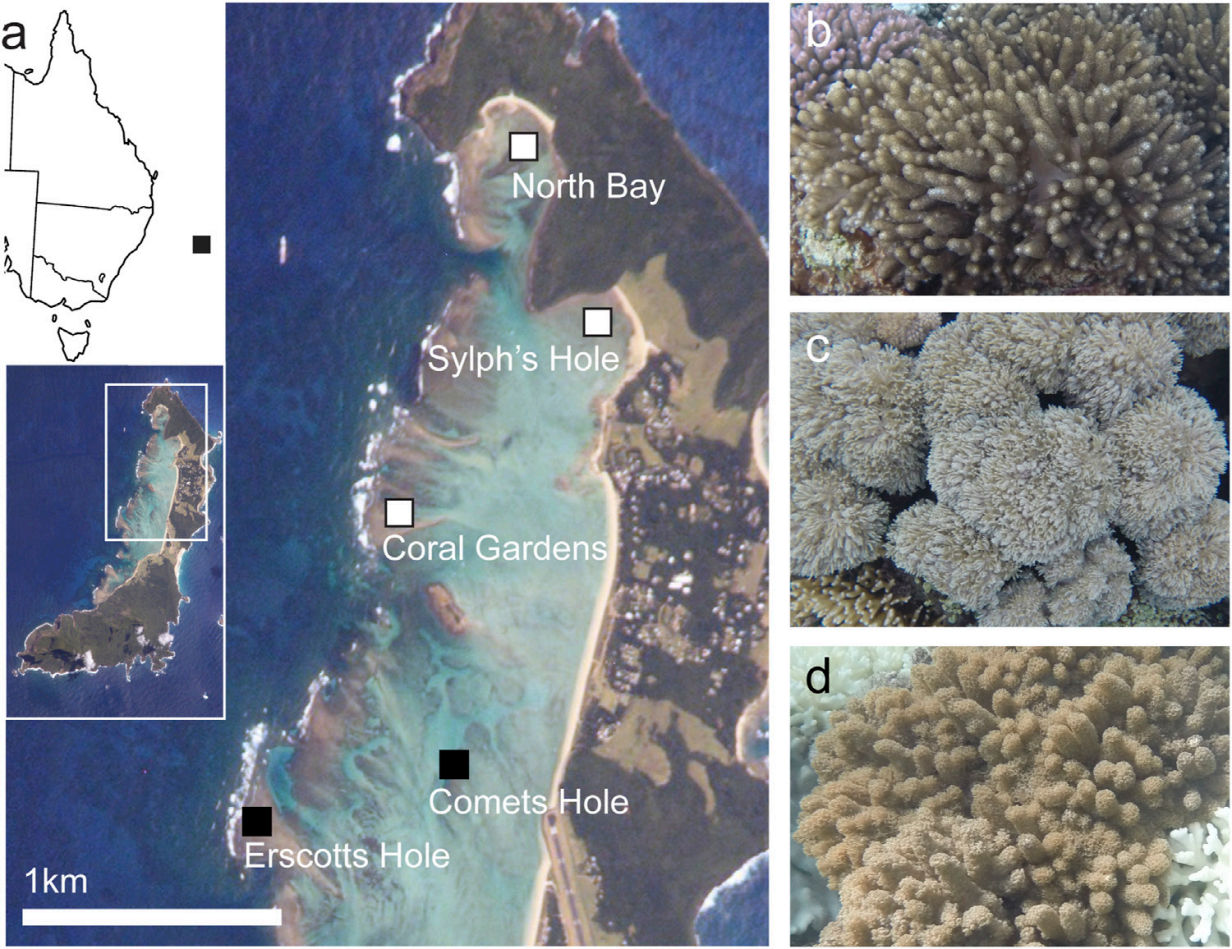
Study sites and species. **(A)** Closeup map of Lord Howe Island, with map of the east coast of Australia and full island map inset. Sites where both surveys and collection were conducted–North Bay, Sylphs Hole, and Coral Gardens–are marked in white with black border, sites where only surveys were conducted–Comets Hole and Erscotts Hole–are marked in black. Lord Howe Island image courtesy of the Image Science and Analysis Laboratory, NASA Johnson Space Center; line map of Australia modified from “Australia states blank. png” by Golbez on Wikimedia Commons. **(B)** *Cladiella* sp. 1, **(C)** *Xenia* cf *crassa*, **(D)** *Cladiella* sp. 2. All coral photos by Rosemary K. Steinberg.

**FIGURE 2:**
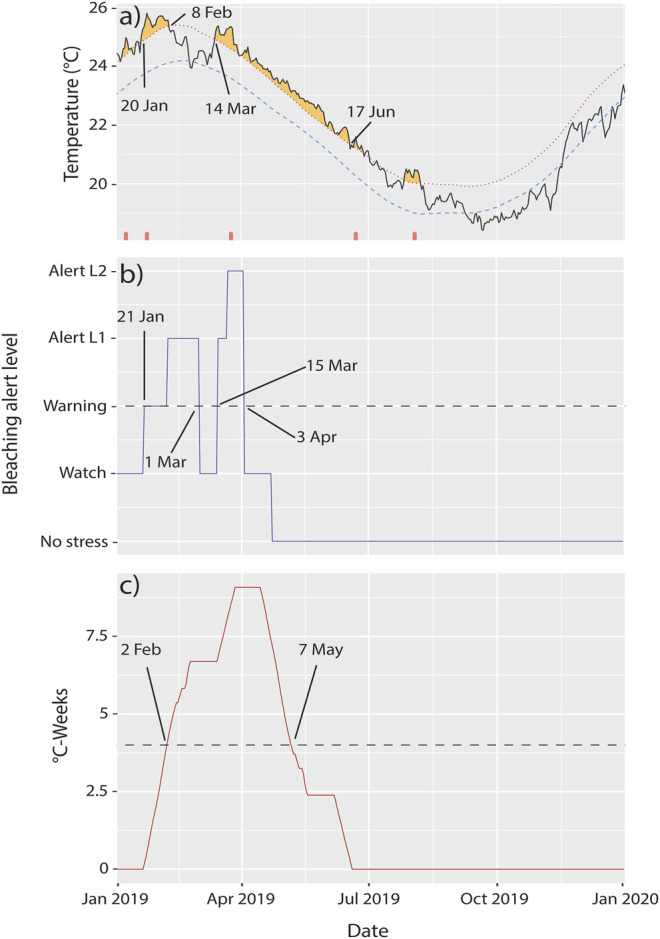
Extreme temperatures at Lord Howe Island in 2019. **(A)** Heatwaves [plot downloaded from the Marine Heatwave Tracker (Schlegel, 2020)], **(B)** Coral Reef Watch bleaching alert levels, and **(C)** degree heating weeks at Lord Howe Island in 2019. The dashed lines in **(B,C)** represent minimum conditions at which bleaching is expected to occur.

Benthic cover was surveyed at five reef sites within Lord Howe Island marine park lagoon—Sylphs Hole, North Bay, Coral Gardens, Erscotts Reef, and Comets Hole (Figure 1A). Three of the five sites, Sylphs Hole, North Bay, and Coral Gardens were further surveyed using 20 m belt surveys and three species of octocorals were sampled (Figure 1A). This sub-sample of sites were chosen for further surveys and sample collections due to variable bleaching prevalence within the hard coral population. While Sylphs Hole experienced the most severe bleaching with 83% of hard corals bleached, North Bay experienced more moderate bleaching with 46% of hard coral cover undergoing bleaching, and Coral Garden’s had the lowest observable bleaching at 16% of hard coral cover bleached (Moriarty et al., in review). These three sites differ in their environmental conditions, with Sylphs Hole highly sheltered and close to shore, North Bay sheltered but near the lagoon edge, and Coral Gardens unsheltered and exposed to wave action (Moriarty et al., in review). CoralWatch bleaching status and degree heating weeks (DHWs) were assessed via the satellite derived ocean temperature monitoring of NOAA Coral Reef Watch and marine heatwave status was assessed using the Marine Heatwave Tracker (NOAA, 2020; Schlegel, 2020) (Figure 2).

### 2.2 Monitoring

Monitoring of coral bleaching was first undertaken during March 13th to 29th, 2019 coinciding with peak summertime sea surface temperatures (Figure 2A), the sites were also re-surveyed 1 month after when bleaching alerts had subsided (April 26^th^–May 2^nd^, 2019, Figure 2B), and again in the southern hemisphere’s spring from October 16^th^ to 31st, 2019. All species of soft cnidarians including *Anthelia* sp., *Xenia* cf *crassa, Xenia* sp., *Cladiella* sp. 1, *Cladiella* sp. 2 sp., *Entacmaea quadricolor,* and *Palythoa* sp. were quantified in photo quadrat and belt surveys. Octocoral cover was assessed in 1 × 1 m quadrat photos at intervals 4, 12, and 16 m along each transect (Nikon Coolpix and an Olympus TG5 in underwater mode). The fixed intervals 4, 12, and 16 m were randomly chosen from a number sheet. All quadrats were photographed on the same side of the transect tape for each transect. Photo quadrats were annotated using CoralNet using 20 fixed points per quadrat (Beijbom et al., 2012). Anemones could not be seen on photo quadrats as they reside within crevices. At Coral Gardens, North Bay, and Sylphs Hole, species composition and colony numbers (abundance) were recorded within 20 × 1 m belt transects (snorkel based transect swims), wherein crevices and overhangs were extensively checked for anemones and octocoral colonies. Visual health status (visually bleached or unbleached) was noted for each surveyed cnidarian. Species not found on transects but seen during travel to and from survey sites or while collecting samples were recorded as off-transect observations.

### 2.3 Collection Methods

Three species of octocorals were sampled and bleaching quantified - *Cladiella* sp. 1, *Xenia* cf *crassa,* and *Cladiella* sp. 2 (Figure 1C.). Octocorals were identified to genus using Fabricius and Alderslade (2001) and *Cladiella* sp. 1 and sp. 2 were identified as different species based on external and spicule morphology (Table 1). *Xenia* cf *crassa* and *Cladiella* sp. 2 were chosen as they were common at all sampling sites and Xeniids have previously been more bleaching susceptible than Alcyoniids (Strychar et al., 2005; Sammarco and Strychar, 2013), while *Cladiella* sp. 1 (Figure 1D) was chosen because bleaching was first observed in this species at Sylphs Hole on 26 March 2019 (*Cladiella* sp. 1 was collected at all sites and monitoring intervals excluding North Bay in March due to logistical constraints).

**TABLE 1:** Summaries of study species morphological features and habitat preferences.

Species	Colony morphology	Polyp morphology	Body retraction	Spicule morphology	Habitat preference
*Cladiella* sp. 1	Lobate. Lobes short and stiff. Brown colour with some reddish or orange tints	Short (<1 mm), retractable polyps. Classic rosette tentacle formation. Polyps are darker than body	Highly retractile. Retracted colonies are the colour of the body (light brown) with small, dark pin-pricks where the polyps are retracted	Spiky, nearly rod-like dumbbell shaped spicules. The central “handle” is small and the “heads” are narrow	Near the bottom of shallow reef on rock edges. Form large, dense aggregations
*Cladiella* sp. 2	Lobate. Lobes long and flexible. Deep brown colour	Short (<1 mm), retractable polyps. Rosette tentacle formation with twisted, curly tentacles	Highly retractile. Stark change in colour to grey when retracted. Retracted polyps are difficult to see, can be made out as dents in the colony surface	Spiky dumbbell shaped spicules. Centre “handle” much larger than *Cladiella* sp. 1 and “heads” are large compared to the “handle”	Near the bottom of shallow reef on rock edges and in reef crevices. Form small aggregations
*Xenia* cf crassa	Pom-pom. Flexible, tube-shaped bodies with long (>3 cm) polyp stalks on the top side only. Tube is light brown, polyp stalks are dark brown, and tentacles are dark brown with reflective blue on the top sides, which can make colonies appear blue or silver from above	Very long polyp stalks (>3 cm) and tentacles (>5 mm). Polyp stalks are thin and densely packed, polyps have feathery tentacles with reflective blue or silver surface	Moderately retractile. Polyp stalks shrink down towards the body and polyps close	Small, flat, disk-shaped spicules	At the top of reef outcrops and on top of dead coral. Form large and expansive mats

Octocoral fragments were collected using Australian Entomological Supplies PTY LTD 12.5 cm blunt-tipped surgical scissors and placed in individual zip-top bags. Ten samples per species of unbleached and ten samples per species of bleached corals were collected at each coral reef site, with all samples collected from different colonies. Where no bleached samples of a species were found within a reef site only unbleached colonies were collected. Samples were minimally handled after collection, kept in individual zip-top bags in a cooler during transport, and handled and transported for as little time as possible to minimize handling, thermal, and light stress. Each sample was split into two portions–one was immediately fixed in a sterile solution of 4% formalin in 3× phosphate buffered saline (PBS) in nuclease free water and kept at 4°C for later DNA extraction. Samples were transferred to a sterile solution of 3× PBS after 10–14 h in fixative and stored at 4°C. The other portion were kept in aquaria on the day of collection within the laboratory at the Lord Howe Island research station (March), at the Lord Howe Island Marine Park Authority boat shed (April/May), or at the facility provided by Blue Lagoon Lodge (October) with aeration until 30 min after sundown to measure maximum quantum yield of photosystem II (PSII) on live colonies, after which they were frozen. Aquaria consisted of 54 L plastic tubs filled partially with unfiltered seawater and kept aerated with use of a bubbler and no source of artificial light. Soft coral fragments were kept separated by keeping them in small, submerged collection jars weighed down with stones.

### 2.4 DNA Extraction, PCR, and Sequencing

A subset of five samples each of healthy *Cladiella* sp.1, sp. 2, and *Xenia* cf *crassa* at Coral Gardens and Sylphs hole during March and October were processed to determine Symbiodiniaceae type profiles using the ITS2 gene. DNA was extracted using a Qiagen QIAamp DNA Mini Kit following the manufacturers tissue protocol (Qiagen, 2016) with the following modifications: tissue was bead-beaten in 180 µl of buffer ATL, and 20 µl of proteinase K was added, after which the tissue was digested at 56°C overnight. Following digestion and washing, DNA was eluted in the provided buffer AE and the buffer was left on the membrane for 10 minutes before centrifugation; the same aliquot of buffer was returned to the membrane for a second time for another 10 minutes and centrifuged. Cladiella sp. 1 DNA was eluted in 200 µl, Xenia cf crassa DNA was eluted in 50 µl, and Cladiella sp. 2 DNA was eluted in 100 µl buffer AE. ITS2 PCR was performed following the protocol from Hume et al. (2018) using SYM_VAR_5.8S2 - SYM_VAR_REV primers. Sequencing was performed by the Ramaciotti Centre for Genomics, UNSW (Sydney, NSW, Australia).

### 2.5 Pulse Amplitude Modulated Fluorimetry

Maximum quantum yield of PSII (F_v_/F_m_) was measured using PAM fluorimetry. Measurements were taken at least 30 min after sunset in full darkness. Point yield measurements were taken using a Walz Diving-PAM fluorimeter (Heinz Walz GmbH, Effeltrich, Germany, March 2019) and a Maxi Imaging-PAM M-series fluorimeter (Heinz Walz GmbH, Effeltrich, Germany, April/May and October 2019). PAM settings were as follows: Diving-PAM for unbleached coral colonies, MI: 8, SI: 8, Sat-width: 0.85, Gain: 2, Damp: 2. Diving-PAM for bleached colonies: MI: 8, SI: 8, Sat-width: 0.85, Gain: 10, Damp: 2. For the Imaging-PAM, settings for all colonies were MI: 8, SI: 8, Sat-width: 0.8, Gain: 12, Damp: 2. Three replicate readings per coral fragment were recorded. When using the Diving-PAM, replicates on individual fragments were achieved by waiting half an hour between runs to allow fragments to become dark-adapted again, and readings were taken from different sections of the sample (Ralph, 2005). With the Imaging-PAM, all replicates were measured at once as multiple readings can be taken together by defining separate areas of interest within the PAM image (Heinz Walz GmbH, 2019). After PAM fluorimetry, all samples were placed in −4°C freezer for up to 2 weeks in the field and transferred to a −20°C freezer in the laboratory in Sydney and kept frozen until processing for protein, Symbiodiniaceae, and chlorophyll concentrations.

### 2.6 Determination of Coral Protein Content, Symbiodiniaceae Concentrations, and Chlorophyll a and c2 Concentrations

Samples were processed as per Steinberg et al. (2021). In summary, octocoral samples were homogenised with reverse osmosis water using an Omni TH Tissue Homogeniser. The resulting slurry was centrifuged and supernatant removed and saved, and this step was repeated. Supernatant protein was quantified against a bovine albumin standard using the Thermo Scientific Coomasie Plus (Bradford) kit and protocol. Symbiodiniaceae were counted using a Neubauer Improved haemocytometer with three haemocytometer fills, two grids per haemocytometer, and five counts per grid, which were averaged, for a total of 6 replicate counts (Steinberg et al., 2021). Chlorophyll was extracted in 100% acetone for 48 h and measured on a VWR UV-6300PC Double Beam Spectrophotometer (see Steinberg et al. (2021) for more information).

### 2.7 Statistical Analysis

All graphs and analyses were completed in R version 3.4.1 (R Core Team, 2013). Plots were created with the package ggplot2 (Wickham, 2011). Differences in percent benthic cover were compared among benthic groups (octocoral, stony coral, algae, seagrass, and abiotic) and sites (Sylphs Hole, North Bay, Coral Gardens, Erscotts Reef, and Comets Hole) using a generalised linear mixed model (GLMM) in the packaged glmmTMB (Brooks et al., 2017). Differences in community composition among sites (Sylphs Hole, North Bay, and Coral Gardens) and monitoring intervals were tested using permutational multivariate analysis of variances (PERMANOVA) with the ‘adonis’ function in the R package Vegan (Oksanen et al., 2019). All zero-only transects were removed, and dispersion was tested using permutest before running the PERMANOVA. Square-root transformation of count data was performed to ensure non-significant differences in dispersion. Pairwise comparisons were performed using the package pairwiseAdonis (Marinez Arbizu, 2020).

Differences in soft coral abundance, Symbiodiniaceae counts, and chlorophyll concentrations per µg protein among sites, monitoring intervals, and visual health status were tested using a GLMM. For abundance, transect number was included as a random variable. For Symbiodiniaceae and chlorophyll the individual that the sample was taken from was included as a random factor, and either protein content or Symbiodiniaceae density was included as an offset (depending on which factor was used for standardisation). Additional offsets for particular models are outlined in the supplemental methods. For specifics on which distribution was used, see the table associated with each analysis (Supplementary Tables S1–S7). Differences in protein content and mean chlorophyll *a* and *c*_*2*_ per Symbiodiniaceae cell among sites, monitoring intervals, and visual health status were tested using two-way ANOVAs as these datasets contained no random factors. As ANOVA requires data to be normal, datapoints that fell outside the theoretical quantiles of the QQ-plot plotted using the car package were removed (Fox and Weisberg, 2019). Differences in photosynthetic yield among monitoring intervals and visual health status was tested using a linear mixed effects model (lmer) from the package lme4 (Bates et al., 2015). Further model details and example models are included in the supplemental methods. All pairwise comparisons on significant GLMM, lmer, and ANOVA analyses were performed using the emmeans package (Lenth et al., 2018).

## 3 Results

### 3.1 Temperatures in the Lord Howe Island Lagoon

During early 2019, Lord Howe Island was affected by three marine heatwaves (Figure 2A). Heat wave properties reported here are duration, intensity (degrees Celsius above the expected time-of-year mean, reported as mean and max intensity), and cumulative intensity (the integral of intensity over the event). The first heatwave began on 31 Dec 2018 and lasted 14 days to 13 Jan 2019, with a mean intensity of 1.3°C, max intensity of 1.67°C, and a cumulative intensity of 18.2°C. The second heatwave began on 20 Jan 2019 and lasted 20 days until 8 Feb 2019, with a mean intensity of 1.61°C, a max intensity of 2.01°C, and a cumulative intensity of 32.15°C. The third began on 14 Mar 2019 and lasted 96 days until 17 June 2019, with a mean intensity of 1.17°C, max intensity of 1.76°C, and a cumulative intensity of 112.48°C (Schlegel, 2020). Cyclone Oma made landfall on the island between the second and third marine heat waves in late February 2019 (Figure 2A). Coinciding with the heatwaves, NOAA Coral Reef Watch bleaching alert levels met or exceeded “Warning” during two time periods, from 21 Jan to 1 Mar 2019 with alerts reaching level 1, and 15 Mar to 3 Apr 2019 with alert levels reaching level 2. In addition, degree heating weeks (DHWs) met or exceeded 4°C-weeks from 2 Feb 2019 to 7 May 2019. Symbiodiniaceae begin to experience stress when DHWs exceed 2 DHWs, bleaching is expected at 4 DHWs, and significant mortality at 8 DHWs (Gierz et al., 2020; Hughes et al., 2018a; Kayanne, 2017, Figure 2C).

### 3.2 Octocoral Morphological Observations

The three species of octocoral chosen for further analysis (*Cladiella* sp. 1, *Xenia* cf *crassa,* and *Cladiella* sp. 2) differ in their morphology, and as morphological differences are known to influence bleaching susceptibility and resilience in stony corals, morphology is detailed in Table 1. Tissue regions with high Symbiodiniaceae density were visually distinguished in all three species by noting their relative colour as they were considerably darker than surrounding non-symbiotic tissues. In the lobed species, Symbiodiniaceae were concentrated on the outer layer of flesh and in the polyps, with the centre of the lobes nearly perfectly white. In *Xenia crassa*, the polyps and polyp stalks appeared to have higher densities of Symbiodiniaceae than the body, with a dark brown colour concentrated in the polyps and a light brown colour in the coral body. Differences in spicule size and density were also evident. Both lobed species had large, densely packed spicules in the centre of the lobes. This was observed by eye and confirmed by the difficulty in cutting through the centre of large lobes, and by spicule masses becoming lodged in the homogenisation mechanism if they were not cut down small enough. Dumbbell shaped spicules were also occasionally found while counting Symbiodiniaceae and were 2–5 × larger than Symbiodiniaceae cells. *Xenia crassa* had much smaller spicules and were extremely easy to cut and homogenise. A few small (1.5 × Symbiodiniaceae size), flat, disk-shaped spicules were observed while counting Symbiodiniaceae (Table 1).

### 3.3 Octocoral and Anemone Cover, Abundance, and Species Composition

**FIGURE 3:**
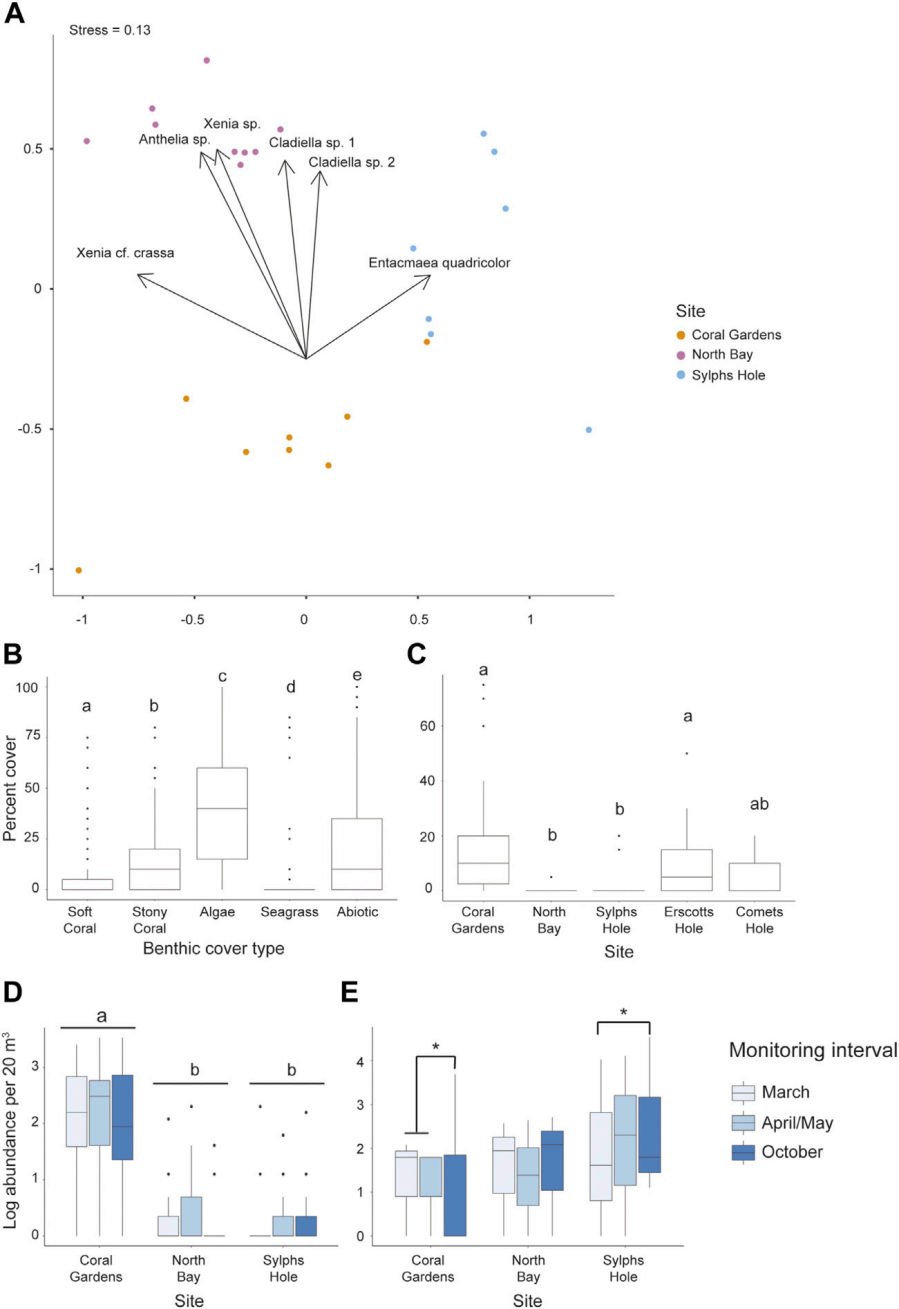
Differences in the percent cover and abundance of octocorals and anemone among benthic groups, sites, and monitoring intervals in the Lord Howe Island lagoon. **(A)** NMDS ordination plot of species composition at each of the sites belt surveyed, Coral Gardens, North Bay, and Sylph’s Hole, **(B)** percent cover from photo quadrats of each benthic group (octocoral, stony coral, algae, seagrass, and abiotic factors) across the lagoon, **(C)** percent octocoral cover from photo quadrats across 5 sites in the lagoon, **(D)** abundance (log) from belt transects of six species of octocoral across three sites in the lagoon, and **(E)** abundance (log) from belt transects of *Entacmaea quadricolor* anemones across three sites and three monitoring intervals in the lagoon. Significance is designated either by different letter group or by a bar. *p*-values are represented as follows—* for *p* < 0.05, ** for *p* < 0.005, and *** for *p* < 0.0005. Where more than one plot is grouped together, the highest *p*-value is reported.

Five species of octocoral (*Cladiella* sp. 1, *Xenia* cf *crassa, Cladiella* sp. 2, *Xenia* sp., *Anthelia* sp.), one anemone species (*Entacmaea quadricolor*), and one zoanthid species (*Palythoa* sp.) were documented within the survey locations at all five reef sites of the Lord Howe Island lagoonal reef system. One additional species of octocoral, *Sarcophyton* sp., was observed at North Bay outside of transects. From the CoralNet quantification of quadrat photos, octocoral cover in the Lord Howe Island lagoon was on average 7 ± 1% SE cover, but was heterogenous between the reef sites within the lagoon (GLMM *p* < 0.0001, min 0%, max 75%, Figure 3B, Supplementary Table S1). Anemone cover was not recorded as anemones occurred primarily in rock and coral crevices and were not visible in photo quadrats. Within the lagoon, octocoral cover (7 ± 1% SE) was significantly different from cover of other benthic groups, with significantly less cover than stony coral (15 ± 2% SE, *p* < 0.0001), algae (39 ± 2% SE, *p* < 0.0001), and abiotic factors (e.g., bare rock or sand, 21 ± 2% SE, *p* < 0.0001), and significantly more cover than seagrass (3 ± 1% SE, *p* < 0.0001, Supplementary Table S1). When examining octocoral cover only across the different sites in the lagoon, there was a significant effect of site, but not of monitoring interval on overall octocoral cover (GLMM *p* < 0.0001, Supplementary Table S1). Octocoral cover was highest at Coral Gardens (17 ± 4% SE) and lowest at North Bay (0.4 ± 0.3% SE; Figure 3C, Supplementary Table S1). Octocoral cover was also significantly higher at Coral Gardens and Erscotts Reef than North Bay or Sylphs Hole, but other site comparisons did not differ (*p* < 0.05, Figure 3C, Supplementary Table S1).

The abundance of octocorals from the belt transects differed significantly among sites (GLMM *p* < 0.0001 Figure 3D), but neither monitoring interval nor the interaction between monitoring interval and site were significant (Supplementary Table S2). Across the lagoon, abundance of all octocoral species was significantly higher at Coral Gardens than North Bay or Sylphs Hole (*p* < 0.0001), but not significantly different between North Bay and Sylphs Hole (Figure 3C, Supplementary Table S2). *Entacmaea quadricolor* abundance differed significantly between monitoring intervals at different sites (GLMM *p* < 0.05, Figure 3A, Supplementary Table S2). At Coral Gardens, octocoral abundance was significantly lower in October than March or April/May (*p* < 0.05). At Sylphs Hole, mean *E. quadricolor* abundance was significantly higher during October compared to abundance recorded during the bleaching event in March (*p* < 0.05). At North Bay abundance did not vary across monitoring intervals. There were no differences in *E. quadricolor* abundances across sites within monitoring intervals.

Sylphs Hole, Coral Gardens, and North Bay had significantly different compositions of octocorals and anemones species (PERMANOVA *p* = 0.001 Figure 3A). *Entacmaea quadricolor, Anthelia* sp., *Cladiella* sp. 1, *Cladiella* sp. 2, and *Xenia* sp. were all abundant at Coral Gardens, while Sylphs Hole was dominated by *Entacmaea quadricolor*, and North Bay had little octocoral or anemone cover, with *E. quadricolor* and *Xenia elongata* being most abundant (Figure 3A, Supplementary Table S2). *Anthelia* sp. and *Xenia* sp. were observed at Coral Gardens, Comets Hole, and Erscotts Reef but not at Sylphs Hole or North Bay.

### 3.4 Symbiodiniaceae Type Profiles

Two Symbiodiniaceae type profiles were identified based on ITS2 sequences, with both *Cladiella* species sharing the type profile C1-C1cy-C42.2-C3-C1cz-C1b and *Xenia* cf *crassa* exhibiting the type profile C1/C42.2-C1b-C1by-C3.

### 3.5 Response of Bleaching Susceptible Species to Heatwaves in the Lord Howe Island Lagoon

#### 3.5.1 Entacmaea Quadricolor Bleaching Response

**FIGURE 4:**
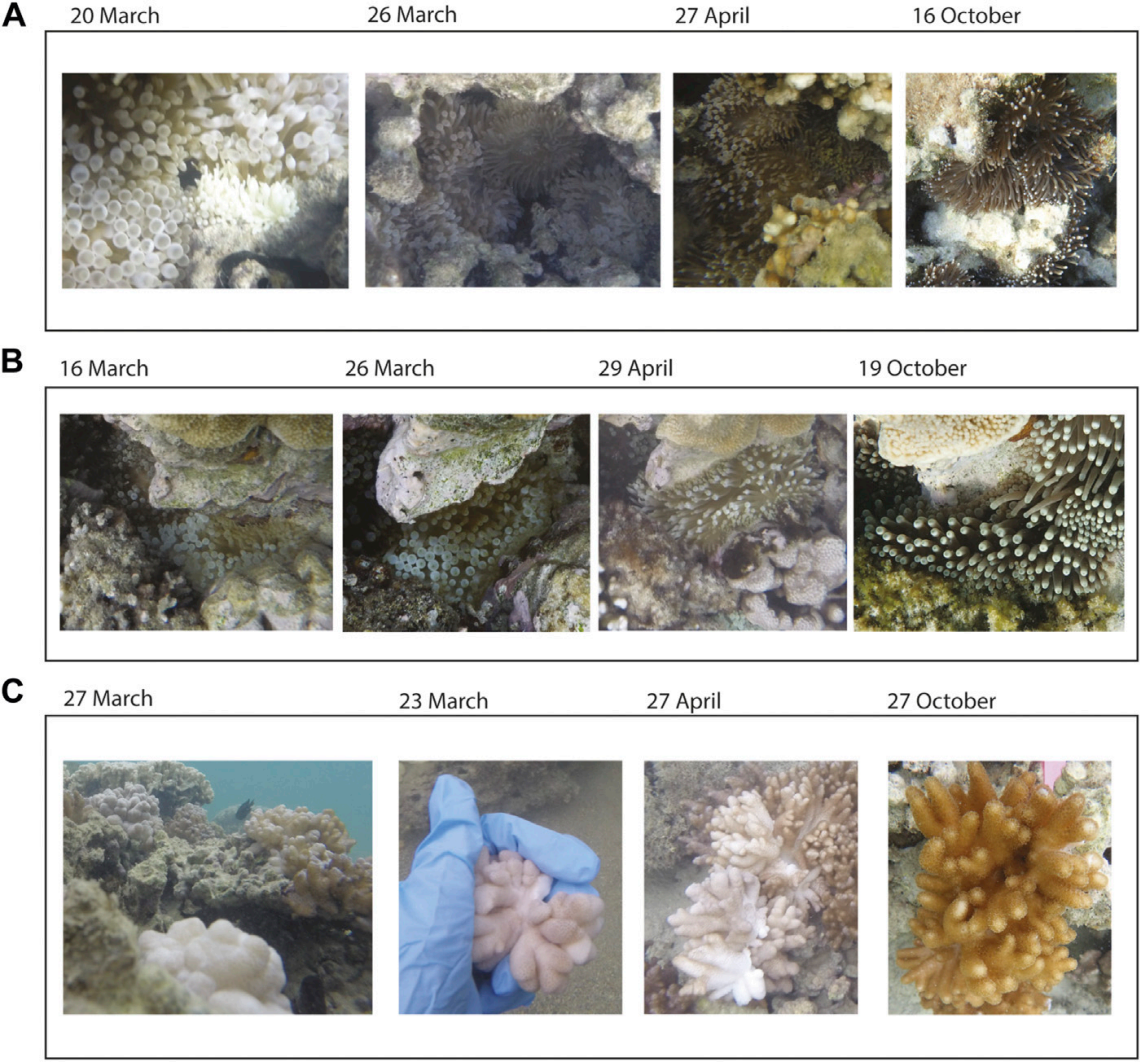
Timelapse photos of *Entacmaea quadricolor* anemones and *Cladiella* sp. 1 octocorals. **(A)** Paled and bleached *E. quadricolor* anemones at Sylphs Hole, **(B)** a single bleached *E. quadricolor* anemone at North Bay, and **(C)** bleached, detached, and semi-detached *Cladiella* sp. 1 at Sylphs Hole. All photos by Rosemary K. Steinberg.

Bleaching or reduced pigmentation (paling) was recorded on transects for the anemone *E. quadricolor* at Sylphs Hole during March and April/May. During March, all anemones recorded were pale or bleached, while during early April we found approximately 60% of anemones were pale and none were observed to be fully bleached (Figure 4A). One bleached *E. quadricolor* anemone was recorded off-transect at North Bay on March 16, 2019, and observed to have partially recovered on 26 March and 29 April 2019 and appeared fully recovered by October 19, 2019 (Figure 4B).

#### 3.5.2 Cladiella Sp. 1 Endosymbiont Bleaching Responses

No bleached *Cladiella* sp. 1 were recorded within the reef surveys but bleached individuals were recorded off-transect at Sylphs Hole during both March and April 2019, with the first bleached colony recorded on 22 March 2019. Bleached colonies were found near, and often mixed within, clusters of unbleached colonies (Figure 4C). Furthermore, seven bleached colonies of *Cladiella* sp. were observed to have detached from the hard substrate and several more colonies were observed to have partially detached (Figure 4C, Supplementary Video S1).

**FIGURE 5:**
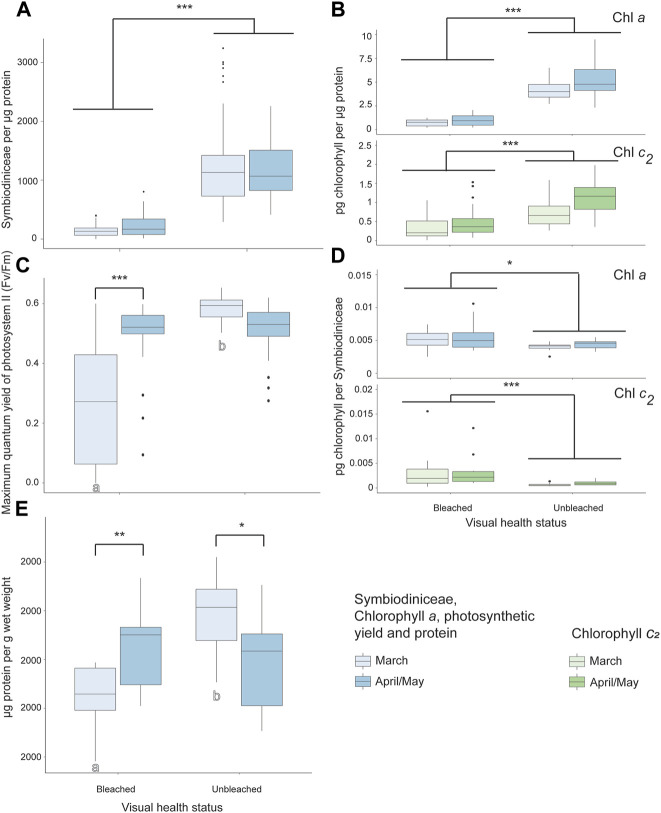
Bleaching responses to a marine heat wave in *Cladiella* sp. 1 at Sylphs Hole Reef in Lord Howe Island lagoon. **(A)** Symbiodiniaceae density per µg protein in visually bleached and healthy *Cladiella* sp. 1 colonies during March and April/May. **(B)** Chlorophyll *a* (above) and *c*_*2*_ (below) densities per µg protein in visually bleached and healthy Cladiella sp. 1 colonies during March and April/May. **(C)** PSII photochemistry of visually bleached and healthy *Cladiella* sp. 1 colonies during March and April/May. **(D)** Chlorophyll *a* (above) and *c*_*2*_ (below) per Symbiodiniaceae cell in visually bleached and healthy *Cladiella* sp. 1 colonies during March and April/May. **(E)** µg protein per g wet weight in visually bleached and healthy *Cladiella* sp. 1 colonies during March and April/May. Significance is designated either by different letter group or by a bar. *p*-values are represented as follows—* for *p* < 0.05, ** for *p* < 0.005, and *** for *p* < 0.0005. Where more than one plot is grouped together, the highest *p*-value is reported. Bars above the boxplots represent monitoring interval differences within health state, and letters below the boxplots represent health state differences within monitoring intervals.

Symbiodiniaceae, chlorophyll *a*, and chlorophyll *c*_*2*_ per µg protein were significantly lower, while chlorophyll *a* and *c*_*2*_ per Symbiodiniaceae were significantly higher, in bleached than unbleached colonies (GLMM *p* < 0.05, Figure 5, Supplementary Table S3). PSII photochemistry was significantly lower during the peak SST temperature of March compared to 1 month after bleaching onset (April/May) in bleached coral colonies of *Cladiella* sp. 1 (*p* < 0.0001). PSII photochemistry was also significantly higher in unbleached than bleached colonies for *Cladiella* sp. 1 during March, though not significantly different in April/May (*p* < 0.0001, Figure 5C, Supplementary Table S3). In March, protein content was significantly lower in bleached coral colonies of *Cladiella* sp. 1 compared to those colonies not found to undergo bleaching (*p* < 0.0001), but in April/May there was no significant difference in protein content between bleached and unbleached colonies (Figure 5D, Supplementary Table S3, two outliers from a total of 118 observations were removed prior to analysis).

#### 3.5.3 Response of Unbleached Cladiella Sp. 1 Colonies

**FIGURE 6:**
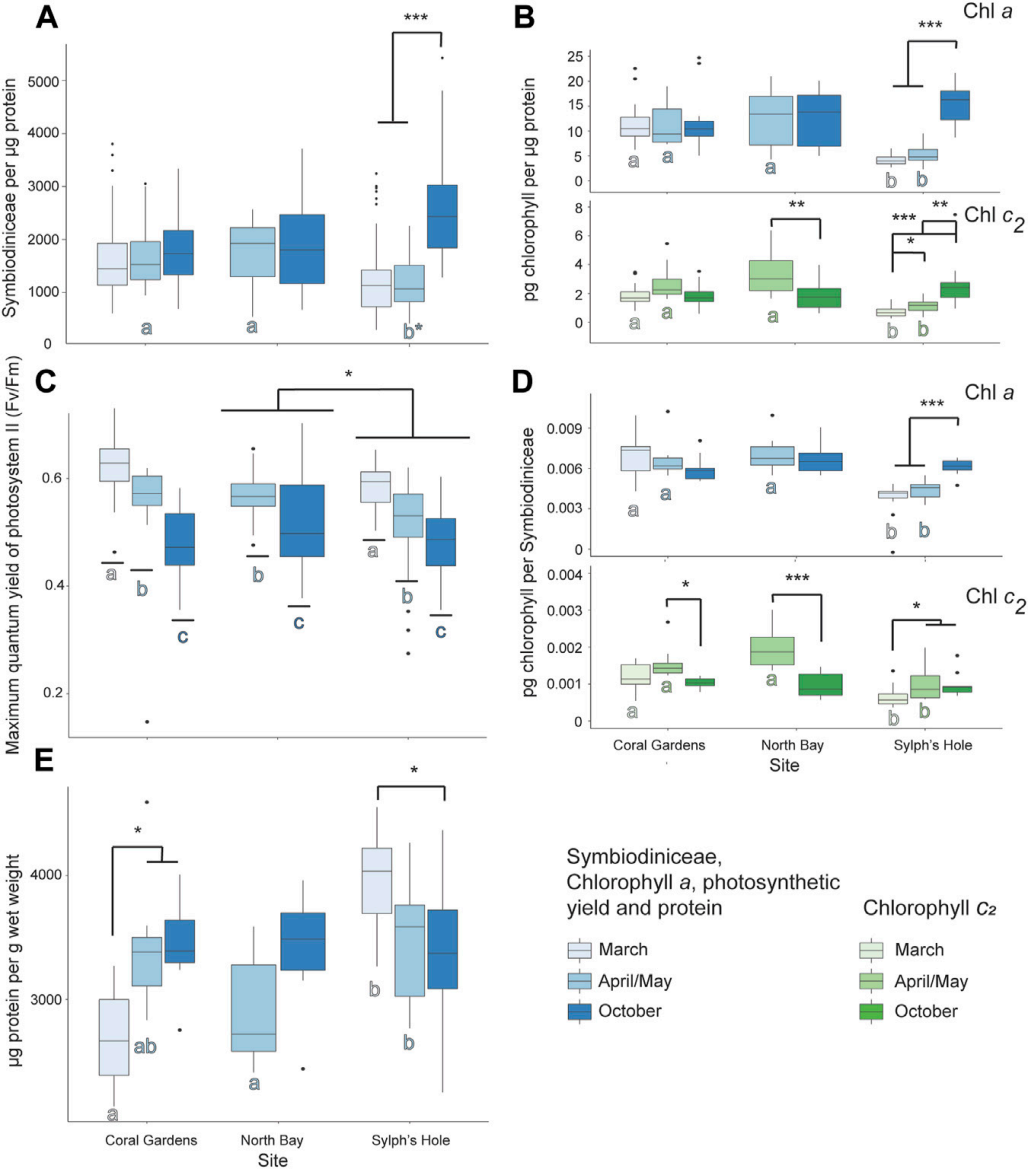
Marine heatwave effects on unbleached *Cladiella* sp. 1 across three reefs in the Lord Howe Island lagoon. **(A)** Symbiodiniaceae density per µg protein in unbleached *Cladiella* sp. 1 colonies during March, April/May, and October. **(B)** Chlorophyll *a* (above) and *c*_*2*_ (below) densities per µg protein in unbleached *Cladiella* sp. 1 colonies during March, April/May, and October. **(C)** PSII photochemistry in visually healthy *Cladiella* sp. 1 colonies during March, April/May, and October. **(D)** Chlorophyll *a* (above) and *c*_*2*_ (below) per Symbiodiniaceae cell in visually healthy *Cladiella* sp. 1 colonies during March, April/May, and October. **(E)** µg protein per g wet weight in visually healthy *Cladiella* sp. 1 colonies during March, April/May, and October. Significance is designated either by different letter group or by a bar. *p*-values are represented as follows—* for *p* < 0.05, ** for *p* < 0.005, and *** for *p* < 0.0005. Where more than one plot is grouped together, the highest *p*-value is reported. Bars above the boxplots represent monitoring interval differences within site, and letters below the boxplots represent site differences within monitoring intervals.

In unbleached *Cladiella* sp. 1 colonies, all measures are reported within site and monitoring interval, as the interaction term in the GLMM was significant (GLMM *p* < 0.05, Figure 6, Supplementary Table S4, two outliers of 271 were removed before protein analysis). In *Cladiella* sp. 1 at Coral Gardens, chlorophyll *c*_*2*_ per Symbiodiniaceae was significantly higher in April/May than October, while protein per wet weight was significantly lower during March than either April/May or October (*p* < 0.05). No other measured variables were significant in *Cladiella* sp. 1 at Coral Gardens (Figure 6, Supplementary Table S4). However, at North Bay, we found both chlorophyll *c*_*2*_ per µg protein and per Symbiodiniaceae in *Cladiella* sp. 1 were significantly higher during March than April (*p* < 0.005, Figure 6, Supplementary Table S4). At Sylphs Hole, concentrations of all measured factors except PSII photochemistry and protein per wet weight were significantly lower in March compared to October (*p* < 0.05, Figure 6, Supplementary Table S4), whereas protein per wet weight was significantly higher at Sylphs Hole during March compared to October (*p* = 0.01, Figure 6E, Supplementary Table S4). Additionally, there were significantly lower concentrations of chlorophyll *c*_*2*_ per µg protein and per Symbiodiniaceae at Sylphs Hole in March compared to April/May (*p* < 0.05), but no differences in other factors (Figure 6, Supplementary Table S4). There were also significantly lower concentrations of all chlorophyll measures at Sylphs Hole than Coral Gardens or North Bay during March, and significantly lower concentrations of all chlorophyll measures and of Symbiodiniaceae per µg protein at Sylphs Hole than Coral Gardens or North Bay during April/May (*p* < 0.001, Figure 6, Supplementary Table S4).

### 3.6 Response of Bleaching Resistant Species to Heatwaves in the Lord Howe Island Lagoon

#### 3.6.1 Response of Unbleached Xenia cf Crassa Colonies

**FIGURE 7:**
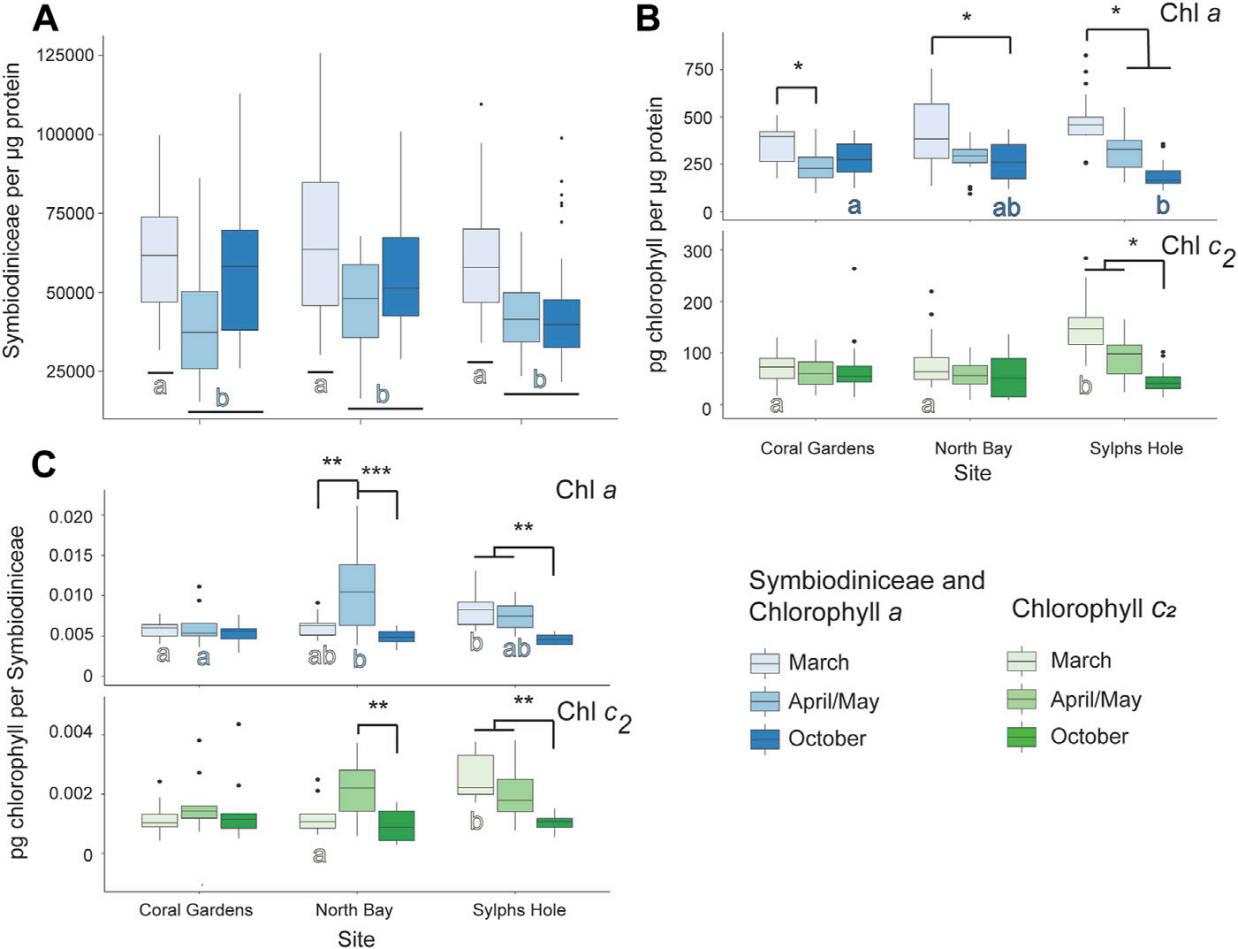
Marine heatwave effects on unbleached *Xenia cf crassa* across three reefs in the Lord Howe Island lagoon. **(A)** Symbiodiniaceae density per µg protein in unbleached *Xenia* cf *crassa* colonies during March, April/May, and October. **(B)** Chlorophyll a (above) and c_2_ (below) densities per µg protein in unbleached *Xenia* cf *crassa* colonies during March, April/May, and October. **(C)** Chlorophyll *a* (above) and *c*_*2*_ (below) densities per Symbiodiniaceae cell in visually healthy *Xenia* cf *crassa* colonies during March, April/May, and October. Significance is designated either by different letter group or by a bar. *p*-values are represented as follows—* for *p* < 0.05, ** for *p* < 0.005, and *** for *p* < 0.0005. Where more than one plot is grouped together, the highest *p*-value is reported. Bars above the boxplots represent monitoring interval differences within site, and letters below the boxplots represent site differences within monitoring intervals.

Symbiodiniaceae per µg protein was significantly higher during the bleaching event in March compared to April/May or October (*p* < 0.05, Figure 7A, Supplementary Table S5). For *Xenia sp*. collected at Coral Gardens, there was significantly higher chlorophyll *a* per µg protein during March compared to April (*p* = 0.04) and no difference in other measured factors. In colonies collected at North Bay, there was significantly higher chlorophyll *a* per µg protein in March compared to October, significantly more chlorophyll a per Symbiodiniaceae in April/May compared to March or October, and more chlorophyll *c*_*2*_ per Symbiodiniaceae during April/May compared to October (*p* < 0.05, Figure 7, Supplementary Table S5). For *Xenia* collected at Sylphs Hole chlorophyll *a* per µg protein was significantly higher during March than April/May or October, and all other chlorophyll measures were significantly higher during March and April/May than October (*p* < 0.05, Figure 7, Supplementary Table S5). During March chlorophyll *a* per Symbiodiniaceae was significantly higher at Sylphs Hole than Coral Gardens, and chlorophyll *c*_*2*_ per µg protein and per Symbiodiniaceae were significantly higher at Sylphs than the other two sites (*p* < 0.05, Figure 7, Supplementary Table S5). During April, chlorophyll *a* per Symbiodiniaceae was significantly lower at Coral Gardens than North Bay (*p* = 0.002, Figure 7C, Supplementary Table S5). During October chlorophyll *a* per µg protein was significantly higher at Coral Gardens than Sylphs (*p* = 0.008, Figure 7B, Supplementary Table S5). Results for PSII photochemistry and protein per wet weight are presented in Supplementary Table S7 and Supplementary Figure S2 but are not discussed further here.

#### 3.6.2 Response of Unbleached Cladiella Sp. 2 Colonies

**FIGURE 8:**
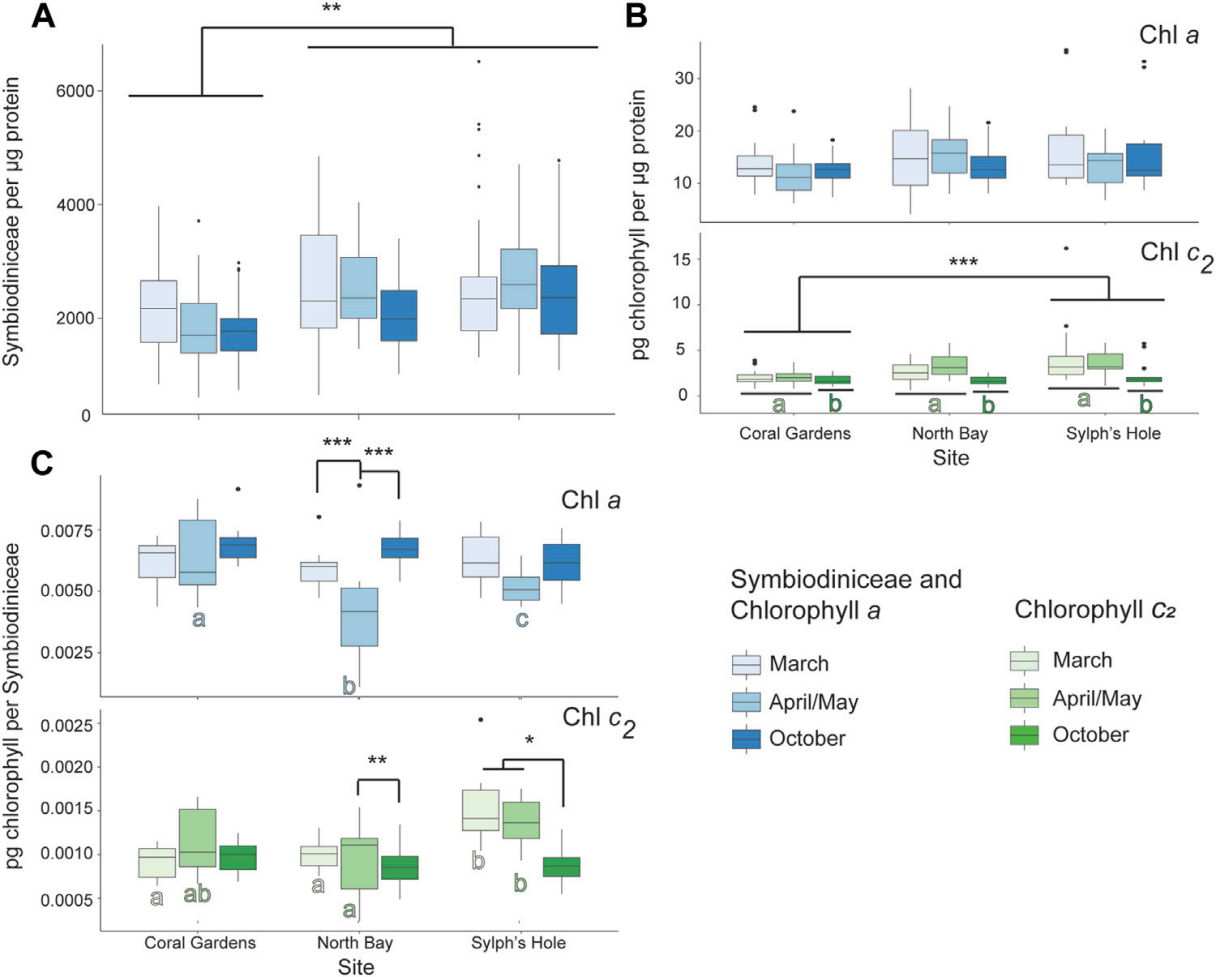
Marine heatwave effects on unbleached *Cladiella* sp. 2 across three reefs in the Lord Howe Island lagoon. **(A)** Symbiodiniaceae density per µg protein in unbleached *Cladiella* sp. 2 colonies during March, April/May, and October. **(B)** Chlorophyll a (above) and c_2_ (below) densities per µg protein in unbleached *Cladiella* sp. 2 colonies during March, April/May, and October. **(C)** Chlorophyll *a* (above) and *c*_*2*_ (below) densities per µg protein in visually healthy *Cladiella* sp. 2 colonies during March, April/May, and October. Significance is designated either by different letter group or by a bar. *p*-values are represented as follows—* for *p* < 0.05, ** for *p* < 0.005, and *** for *p* < 0.0005. Where more than one plot is grouped together, the highest *p*-value is reported. Bars above the boxplots represent monitoring interval differences within site, and letters below the boxplots represent site differences within monitoring intervals.

Symbiodiniaceae density was significantly lower at Coral Gardens than the other two sites, while chlorophyll *c*_*2*_ was significantly lower at Sylphs Hole than Coral Gardens (*p* < 0.05, Figure 8, Supplementary Table S6). Additionally, chlorophyll *c*_*2*_ density was significantly lower during October than the other two monitoring intervals (*p* < 0.05, Figure 8, Supplementary Table S6). At Coral Gardens, Symbiodiniaceae and chlorophyll densities were not significantly different between monitoring intervals. At North Bay, corals had significantly less chlorophyll *a* per Symbiodiniaceae during April/May compared to March and October (*p* < 0.0001, Figure 8C, Supplementary Table S6). Conversely, there was significantly more chlorophyll *c*_*2*_ per Symbiodiniaceae during April/May compared to October (*p* = 0.001, Figure 8, Supplementary Table S6). At Sylphs Hole, *Cladiella* sp. 2 had significantly less chlorophyll *c*_*2*_ per Symbiodiniaceae during October than March or April/May (*p* < 0.05, Figure 8, Supplementary Table S6). During March chlorophyll *c*_*2*_ per Symbiodiniaceae was significantly higher at Sylphs Hole than either Coral Gardens or North Bay (*p* < 0.0005, Figure 8, Supplementary Table S6), while during April chlorophyll *c*_*2*_ was significantly higher at Sylphs Hole than North Bay only (*p* = 0.004, Figure 8, Supplementary Table S6). Results for PSII photochemistry and protein per wet weight are presented in Supplementary Table S8 and Supplementary Figure S2 but are not discussed further here.

## 4 Discussion

On Lord Howe Island marine park lagoonal reefs, species-specific octocoral bleaching responses were evident during the 2019 heatwaves. Here, we surveyed five octocoral species and one anemone species within the Lord Howe Island lagoon and found only two of the examined species bleached. While *Cladiella* sp. 1 and *Entacmaea quadricolor* both bleached, only *Cladiella* detached from the substrate and likely underwent mortality. Though other studies have recorded bleaching and mortality of octocorals, none have reported this type of behaviour. The other four octocoral species surveyed, including a congeneric *Cladiella* species, appeared resistant to the heatwaves and were not observed to undergo bleaching or mortality, during or after, the marine heatwave event. In this study we provide extensive evidence for differential bleaching thresholds of the octocoral species within the Lord Howe Island lagoonal reef system, with species in the same genus exhibiting differential susceptibilities. Previous studies of octocorals in the Great Barrier Reef revealed similar variability in bleaching responses within and among taxa, and this study provides the southernmost recording of bleaching variability in Australian octocorals (Goulet et al., 2008a).

### 4.1 Coral Cover, Abundance, and Species Composition at Lord Howe Island Lagoonal Reefs

Octocorals are important members of tropical and subtropical reefs worldwide. Across the Lord Howe Island lagoon, octocorals are the fourth most abundant biotic benthic group, with mean octocoral cover up to 17% on individual reefs and five species commonly found across sites. Octocoral cover and abundance was highest at the reef edge sites, Coral Gardens and Erscotts Hole, which also had two species (*Anthelia* sp. and *Xenia* sp.) not recorded at the near-shore study sites, North Bay and Sylphs Hole. This is equivalent to cover found in shallow water of the Great Barrier Reef, the Philippines, and Indonesia, higher than reported on shallow water coral reef ecosystems of the Kimberly, Hong Kong, and the lagoons of the Chagos Archipelago, and lower than reported in Guam and southern Taiwan (Dai, 1993; Fabricius and De’ath, 2001; Sheppard et al., 2008; Slattery et al., 2008; Yeung et al., 2014; Baum et al., 2016; Bryce et al., 2018; Lalas et al., 2021). On the Great Barrier Reef, inner-shelf shallow reef (0–5 m) species richness ranged from 5–8 species and octocoral cover ranged from 15 to 20%, consistent with the highest cover we found at Lord Howe Island, and much higher than cover at our near-shore sites (Fabricius and De’ath, 2001). In the same vein, Philippine species richness ranged from one to eight species, while cover ranged from 0–19% (Lalas et al., 2021). In Jakarta Bay, Indonesia, octocoral cover increased with human-induced eutrophication, with cover averaging 13 ± 6% in near-city waters (Baum et al., 2016). For coral reefs in the shallow Kimberly region of Western Australia, intertidal octocoral cover ranged from 0 to 7%; In the Chagos Archipelago, lagoonal octocoral cover was only 2.65 ± 0.72% (Sheppard et al., 2013); and in the turbid and wave exposed waters of Hong Kong, octocorals were totally absent from very shallow waters (0–1 m) (Yeung et al., 2014; Bryce et al., 2018). Though Lord Howe Island octocoral cover did reach as high as 17% on some reefs, this is still lower than found on several Western Pacific reefs, with cover of individual species of *Sarcophyton, Lobophyton,* and *Sinularia* reaching up to 10% cover on shallow reefs of Taiwan, and mean soft coral cover in the shallows of Western Guam as high as 75% (Dai, 1993; Slattery et al., 2008). As such, octocorals are relatively abundant on eastern Australian reefs, and make up an important component of the benthic community of the Lord Howe Island marine park lagoon.

### 4.2 Response of Bleaching Susceptible Species to Heatwaves in the Lord Howe Island Lagoon

Our study of octocoral bleaching was complemented by a study of stony corals at the same sites and times (Moriarty et al., in review)^1^. While we observed bleaching in only one soft coral and in the only observed anemone species, the parallel study recorded severe bleaching in the four most abundant stony coral species, minimal bleaching recorded in one other common species, and occasional bleaching in several rare species, with up to 83% of stony coral colonies bleaching at the most affected site (Moriarty et al., in review). At nearshore reefs in the Great Barrier Reef (within 20 km of shore), octocorals were less susceptible than stony corals, with only 30% of octocoral cover lost compared to 59% of stony coral cover lost post-bleaching (Thompson and Dolman, 2010). As such, octocorals appear to be less susceptible to bleaching than stony corals in eastern Australian waters. Similar patterns were found across the Caribbean (Lasker, 2003; Dias and Gondim, 2016; Goulet et al., 2017; Mccauley et al., 2018; Cerpovicz and Lasker, 2021). On the other hand, octocorals in Sesoko Island, Japan were more susceptible to bleaching than stony corals, with overall octocoral cover dropping from 34.4 to 0.2% (Loya et al., 2001). Similarly, octocorals were more susceptible than stony corals in Sodwana Bay, South Africa, though the bleaching event was relatively mild (Floros et al., 2004). This suggests that there are differences in species’ susceptibility or environmental conditions between these sites driving the range of stony and octocoral responses observed. Our study from eastern Australia, together with the majority of previous findings from Australia and the Caribbean, suggests that octocorals may be generally more resistant than stony corals to bleaching, but further research is needed over a greater area of the globe (Lasker, 2003, 2005; Drohan et al., 2005; Goulet et al., 2008a, Goulet et al., 2008b, 2017; Baker et al., 2015; Mccauley et al., 2018, 2020; Ramsby and Goulet, 2019; Cerpovicz and Lasker, 2021).

Though stony corals have relatively clear taxonomic and morphological patterns in bleaching susceptibility, octocoral studies provide little evidence for such patterns. In stony corals, branching morphologies are generally considered “losers” while massive or boulder morphologies are considered “winners” in regards to coral bleaching event outcomes (Hueerkamp et al., 2001; Loya et al., 2001; Sebastián et al., 2009). In Caribbean gorgonian octocorals, biogeochemical composition and morphology likely affect bleaching susceptibility and may confer some protection from heat-induced bleaching compared to stony corals inhabiting the same reefs, but we know little of patterns of susceptibility in Alcyonacean octocorals (Baker et al., 2015; Goulet et al., 2017; Mccauley et al., 2018). In this study, congeneric octocorals with similar morphologies displayed variable bleaching responses, as has also been found in bleaching events across the globe (Loya et al., 2001; Goulet et al., 2008a; Slattery et al., 2008; Prada et al., 2010). For example, in Sesoko Island, Japan, two lobate species of octocoral in the same family, Alcyoniidae, were a major contributor to overall reef cover, *Lobophyton* sp. and *Sinularia* sp. (Fabricius and Alderslade, 2001; Loya et al., 2001). Though both species were significantly affected by bleaching, *Sinularia* sp. was more resistant and replaced *Lobophyton* sp. as the dominant octocoral on the impacted reefs (Loya et al., 2001). Similarly, in four reefs across southern Puerto Rico, heat induced bleaching ranged from 0 to 90%, with three species resistant to bleaching pressure (Prada et al., 2010). Of those species, one unbleached (*Eunicea* sp.) and the most highly impacted (*Muricea* sp.) are both in the family Plexauridae and share similar branching morphologies (Prada et al., 2010). Even within species bleaching susceptibility can be variable, with bleached octocorals on the Great Barrier Reef found adjacent to unbleached conspecifics (Goulet et al., 2008a), which was also observed in the present study. Interestingly, the Caribbean octocoral *Briarium asbestinum* has two distinct growth forms, encrusting and branching, which exhibited differential responses to experimental heating, suggesting that the impacts of morphology on octocoral stress response needs to be investigated further (Ramsby and Goulet, 2019). Symbiodiniaceae types hosted by each coral species may also affect bleaching susceptibility as sensitivity to thermal stress can vary between Symbiodiniaceae genera and species (Rowan et al., 1997; Goulet et al., 2005, 2017), but we found that both *Cladiella* sp. 1 and sp. 2 share a type profile and all three species were dominated by Cladocopium C1. This is similar to what was found previously in the Great Barrier reef, where the dominant resident symbiont type did not explain bleaching susceptibility in octocorals (Goulet et al., 2008a; Goulet et al., 2008b). Taken together, it appears that bleaching susceptibility of octocorals cannot be predicted simply by family, genera, growth form, or symbiont type profile; and may instead be influenced by species- and environment-specific factors.

*Entacmaea quadricolor* anemones are important habitat for many species of anemonefish, but are susceptible to bleaching across their range, impacting both anemones and their mutualists (Chadwick and Arvedlund, 2005; Hill and Scott, 2012; Huebner et al., 2012; Thomas et al., 2015; Scott and Dixson, 2016; Frisch et al., 2019). We observed bleaching of the *E. quadricolor* anemone in the Lord Howe Island lagoon, with fairly rapid visual recovery of ∼40% of anemones within a month of the end of the bleaching warnings/alerts. Previous laboratory bleaching studies found that *E. quadricolor* anemones bleached when exposed to as little as 1°C above the average summer temperature, 27°C, with bleaching apparent as little as 3 days post exposure (Hill and Scott, 2012). A second study found that *E. quadricolor* lost 90% of their endosymbiotic algae under increased irradiance at 28.5°C, but began to recover within 25 days of the end of experimental heating (Hill et al., 2014). *In-situ*, one individual *E. quadricolor* anemone took from 3 to 5 months to recover from bleaching, with recovery time increasing in the second year of bleaching (Hayashi and Reimer, 2020). Interestingly, anemone density at the most impacted site at Lord Howe Island increased over the monitoring intervals. It is unlikely the anemones had reproduced over the duration of our study, however, it is possible they became more visible following the end of the heatwave. Anemones retract when bleached (Hill et al., 2014), and it is possible that bleached anemones were retracted and so overlooked during belt surveys in March and April/May. Because bleaching can cause mortality in anemones and can have severe consequences for mutualistic anemonefish, continued monitoring of this population is warranted (Jones et al., 2008; Thomas et al., 2015; Howell et al., 2016; Scott and Dixson, 2016; Scott and Hoey, 2017; Frisch et al., 2019).

Reduction in maximum quantum yield of PSII, protein content, Symbiodiniaceae, and chlorophyll are all consequences of bleaching, and they do not all recover at the same rate. Previous work in anemones found that PSII photochemistry recovered relatively quickly post-bleaching, with incomplete recovery only 4 days after temperatures were returned to normal (Hill and Scott, 2012). This result is consistent with rapid recovery of PSII photochemistry observed in the current study. We also found that protein content recovered quickly, in contrast to protein content in bleached *Lobophytum compactum*, which was reduced during and after bleaching for at least 8 months (Michalek-Wagner and Willis, 2001b). A study on Mexican stony corals found that recovery time for Symbiodiniaceae density differed greatly between species, with some recovering fully in as little as 6 weeks (Grottoli et al., 2014). As in the current study, bleached *Montastrea annularis* also had significantly lower protein content and significantly higher chlorophyll *a* per symbiont than their unbleached counterparts (Fitt et al., 1993). In *Acropora aspera* colonies that had not experienced protective pre-bleaching stress (sub-bleaching heat stress that induces thermal tolerance), there were similar patterns to what was found in *Cladiella* sp. 1 (Ainsworth et al., 2016; Bonesso et al., 2017). After 16 days of heat stress, there was a marked reduction in maximum quantum yield of PSII, a steady decrease in Symbiodiniaceae concentrations, and a decrease followed by an increase in chlorophyll *a* per Symbiodiniaceae (Bonesso et al., 2017). This increase in chlorophyll content may be due to decreased competition between Symbiodiniaceae for nutrients in bleached tissue, as chlorophyll concentration is an indicator of nutrient status (Rees, 1991; Fitt et al., 1993), though an increase in available nitrogen in the water could also increase chlorophyll concentrations (Grover et al., 2002). Bleaching can have long-reaching effects on octocoral population health, including reduced growth, fecundity, sperm motility, and recruitment for years after a bleaching event (Michalek-Wagner and Willis, 2001a). As such, recovery of this species may be hindered by the long-term effects of elevated lagoon temperatures.

Heat stress can also impact corals without causing visual bleaching. Often, bleaching is defined as reduction in Symbiodiniaceae density or chlorophyll (Fitt and Warner, 2001), but this is not always clear in the field as is the case at Lord Howe Island. In the lagoon, visually unbleached *Cladiella* sp. 1 colonies at the warmest and most sheltered site (Sylphs Hole; Moriarty et al., in review) had lower Symbiodiniaceae and chlorophyll concentrations during the heatwave and may have been impacted even though they did not appear bleached to the naked eye. During a bleaching event at Magnetic Island, QLD, Australia in 1994, unbleached *Acropora formosa* had lower concentrations of Symbiodiniaceae and chlorophyll during peak temperatures than during recovery, suggesting that colonies were stressed even though bleaching was not visible (Jones, 1997). Additionally, heat stress that does not result in bleaching can still lead to other adverse health effects. Colonies of *Acropora aspera* exposed to heat stress 2°C below the bleaching threshold had significantly reduced green fluorescent protein fluorescence, reduced skeletal calcification, and reduced healing ability after injury, and nearshore colonies of the Caribbean corals *Siderastrea siderea* and *Pseudodiploria strigosa* have experienced reduction in growth rates across bleaching and non-bleaching time periods (Bonesso et al., 2017; Baumann et al., 2019). As such, understanding whether unbleached corals were impacted by heat stress can help us predict recovery time of reefs after marine heatwaves.

### 4.3 Response of Bleaching Tolerant Species to Heatwaves in the Lord Howe Island Lagoon

Xeniids are fast growing shallow reef species that are highly successful colonisers and occasional invaders, but have previously been found to be susceptible to bleaching (Benayahu and Loya, 1985; Strychar et al., 2005; Goulet et al., 2008a; Wood and Dipper, 2008; Sammarco and Strychar, 2013; Ruiz Allais et al., 2014; Checoli et al., 2018). Interestingly, at Lord Howe Island, *Xenia* cf *crassa* appears resistant to heat induced bleaching pressure in the lagoon. This is in stark contrast to patterns observed in Xeniid corals from the Great Barrier Reef during the 1998 bleaching event, where Xeniid colonies bleached and died within a few days of the onset of bleaching, while Alcyoniids survived for long periods (Goulet et al., 2008a). In laboratory studies, *Xenia* sp. released Symbiodiniaceae cells and experienced host apoptosis at lower temperatures than either *Sarcophyton ehrenbergi* or *Sinularia* sp., both Alcyoniids (Strychar et al., 2005; Sammarco and Strychar, 2013). Additionally, *X. crassa* host cells became apoptotic at lower temperatures than Symbiodiniaceae, suggesting that host cells of *Xenia* sp. are more susceptible to bleaching stress than the Symbiodiniaceae cells (Sammarco and Strychar, 2013). As host cell responses were not measured during the present study, examination of host tissues during natural heatwaves would be beneficial to our understanding of *Xenia* spp. bleaching responses. Interestingly, the control temperature for both studies (28°C) was equal to the bleaching temperature during the Lord Howe Island bleaching event, suggesting that although *Xenia* sp. may be highly susceptible to bleaching once temperatures exceed their bleaching threshold, this was not exceeded at Lord Howe Island. Additionally, repeated heat stress can cause resistant octocoral species to experience bleaching, as reported in *Sinularia polydactyla* in Guam, which started as a “winner” during early bleaching events, but became a “loser” after experiencing six consecutive years of increased ocean summer temperatures (Slattery et al., 2019). As such, although *Xenia* cf *crassa* had increased Symbiodiniaceae and chlorophyll concentrations during the increased lagoon temperatures in this study, if future bleaching events are warmer these corals may be negatively affected.

Corals can be affected by cold stress as well as heat stress. As Lord Howe Island is subtropical, winter temperatures can reach as low as 17.5°C, which may be stressful for some species. Unlike either *Cladiella* sp. 1 or *Xenia* cf *crassa*, there were no strong effects of increased lagoon temperatures on *Cladiella* sp. 2*.* Instead, only chlorophyll *c*_*2*_ content was reduced in October, when temperatures were lowest. This is opposite to what was previously found in manipulative non-bleaching heat stress of Caribbean gorgonian octocorals, where heat stress did not reduce Symbiodiniaceae density, but did reduce chlorophyll concentrations (Goulet et al., 2017), and where chlorophyll *a* and *c*_*2*_ per Symbiodiniaceae cell were higher in winter compared to summer temperatures (Mccauley et al., 2018). Previous work in stony corals found that in cold water, *Acropora aspera* and *Montipora digitata* chlorophyll *a* concentrations were increased, and *Acropora yongei* chlorophyll content was not affected by temperature (Saxby et al., 2003; Roth et al., 2012; Nielsen et al., 2020). *Cladiella* sp. 1 may reduce chlorophyll *c*_*2*_ in cold water as they are acclimated to the cool winter temperatures of the subtropical island and are not experiencing stress. Unfortunately, little is known about the heat or cold tolerance of this genus. As *Cladiella* sp. 1 was the only octocoral species to bleach during the 2019 heatwaves at Lord Howe Island, it is evident that there is variation in susceptibility within this genus. *Cladiella* spp. are widespread throughout the world, with populations in Australia, Asia, the Pacific Islands, Africa and the Red Sea, and are moderately common in coastal waters, and as such a greater understanding of the variation in susceptibility to bleaching in this genus would have worldwide implications (Fabricius and Alderslade, 2001).

### 4.4 Conclusion

We found that across octocoral and anemone species the impacts of marine heatwaves in the Lord Howe Island lagoon are quite variable, with evidence of a range of thermal susceptibility and resistance in these members of the reef ecosystem. Morphological traits are recognised as important determinants of bleaching resistance in hard corals, but no such patterns have yet become evident in studies of non-gorgonian octocorals (Conti-Jerpe et al., 2020). Within soft cnidarians of the Lord Howe Island lagoon, *Xenia* cf *crassa* and *Cladiella* sp. 2 were resistant to bleaching, while *Cladiella* sp. 1 and *Entacmaea quadricolor* were susceptible during this particular series of marine heatwaves. As marine heatwaves increase in their duration, intensity and frequency (Oliver et al., 2018), it may be that octocorals become a more abundant component of the world’s southern-most coral reef ecosystems (Van Hooidonk et al., 2016). Repeated bleaching events can turn octocoral winners into losers, as such, if marine heatwaves continue to affect the Lord Howe Island lagoon, current resistant species may become susceptible. In order to minimise ecological and social impacts of warming events, it is necessary to have a better understanding of susceptibility, resistance, and resilience across this delicate ecosystem. If heatwaves continue to affect the Lord Howe Island lagoon, shifts in community structure may occur as abundant yet sensitive species bleach and die, and the less common yet tolerant species, such as *Xenia* cf *crassa* and *Cladiella* sp. 2, remain unaffected, as has previously been recorded in other reefs (Norström et al., 2009; Cerpovicz and Lasker, 2021). As such, understanding the possible future of Lord Howe Island reefs under increasingly frequent bleaching events should include assessment of both soft and stony corals, and should avoid generalisation. Effective assessment requires understanding of the susceptibility of individual species**.**

## Data Availability

The datasets presented in this study can be found in online repositories. The names of the repository/repositories and accession number(s) can be found below: Mendeley Data, DOI: 10.17632/wvcs8h8yfg.2.

## Footnotes

1 Moriarty, T., Leggat, W., Heron, S., Steinberg, R. K., Gudge, S., and Ainsworth, T. D. Bleaching, Mortality and Lengthy Recovery on the Coral Reefs of Lord Howe Island. The 2019 marine Heatwave Suggests an Uncertain Future for High-Latitude Ecosystems. PLOS Climate.
