# Supplementary material for: Bleaching Susceptibility and Resistance of Octocorals and Anemones at the World’s Southern-Most Coral Reef
Source: Front Physiol. 2022 May 19;13:804193. doi: 10.3389/fphys.2022.804193 (PMC9161773; doi:10.3389/fphys.2022.804193)
Supplement: Supplementary file 1 [file Table1.DOCX]

**Supplemental information**

**Methods**

Example code for analysis of percent cover, abundance, Symbiodiniaceae per µg protein, chlorophyll per µg protein, chlorophyll per Symbiodiniaceae cell, F_v_/F_m_, and µg protein per wet weight. All examples use code for *Cladiella* sp. 1.

Percent cover between groups:

fit <- glmmTMB(count~group +(1|trip) +(1|site) + (1|random), family = nbinom1, data = group.counts.long.no.OI)

where count is the number of points counted for each each group in each photo quadrat, group is the benthic group (abiotic, algae, hard coral, etc.), trip is monitoring interval, site is sampling site, and random is defined as transect within site, as follows:

group.counts.long$random <- paste(group.counts.long$site,group.counts.long$transect,sep = "-")

Percent cover of octocorals between sites and monitoring intervals:

fit2 <- glmmTMB(count ~ trip+site + (1|random), family = nbinom1, data = count.softies)

where count is the number of soft corals recorded in each photo quadrat and the rest of the factors are defined as above.

Abundance of octocorals and anemones:

fit1 <- glmmTMB(Abundance~site*trip + (1|random), family = nbinom1, data = surv.soft)

where Abundance is the number of either octocorals or anemones counted during 20x1m belt surveys and all other factors are defined as above.

Species composition at each reef site surveyed:

surv.adonis <- adonis(sqrt.surv.numeric.no0 ~ surv.predictors.no0$site*surv.predictors.no0$trip,permutations = 999, method = "bray")

where sqrt.surv.number.no0 is the square root of abundance of each species surveyed with any all 0 rows removed, and site and trip are defined as above.

Symbiodiniaceae per µg protein for bleached vs. healthy:

fit2 <- glmmTMB(sum.count ~ health*trip + (1|Number) + offset(log(sum.proportional)), family = poisson, data = zoox.clad1.syl)

where sum.count is the sum of all counts within a haemocytometer grid, health is health state (healthy or bleached), Number is the individual sample counts were taken from, and sum.proportional is:

zoox.clad1.syl$sum.proportional <- zoox.clad1.syl$protein*zoox.clad1.syl$gridvol*5/zoox.clad1.syl$dilution

where protein is µg protein per ml, gridvol is the volume of one grid square, and dilution is the dilution factor for counting. 5 was included as each sum is of 5 grid squares, allowing the glmmTMB analysis to average the counts.

Symbiodiniaceae per µg protein for unbleached across three sites:

fit2 <- glmmTMB(sum.count ~ trip*site + (1|Number) + offset(log(sum.proportional)), family = poisson, data = zoox.clad1.he)

where all factors are defined as above.

Chlorophyll per µg protein for bleached vs. healthy:

fitlog <- lmer(log(chlorophyll.x)~trip*health + (1|Sample.name) + offset(log(protein)), data = chlclad1.syl)

where chlorophyll.x is chlorophyll *a* or *c_2_* per ml, Sample.name is the individual the samples came from, and all other factors are as defined above.

Chlorophyll per µg protein for unbleached across three sites:

fitlog <- lmer(log(chlorophyll.s)~trip*site + (1|Sample.name) + offset(log(protein)), data = chlClad1.he)

where all factors are as defined above.

Chlorophyll per Symbiodiniaceae for bleached vs. healthy:

fitlog <- aov(log(chl.x.per.zoox)~trip*health, data = all.data.clad1.syl)

where all factors are as defined above.

Chlorophyll per Symbiodiniaceae for unbleached across three sites:

fitlog <- aov(log(chl.x.per.zoox)~trip*site, data = all.data.clad1.he)

where all factors are as defined above.

F_v_/F_m_ for bleached vs. healthy:

fit1 <- lmer(YII ~ trip *health +(1|number), data = pam.clad1.syl)

where YII is F_v_/F_m_, and all other factors are as defined above.

F_v_/F_m_ for unbleached across three sites:

fit1 <- lmer(YII ~ trip *site +(1|number), data = pam.clad1.he)

where all factors are defined as above.

Protein per wet weight for bleached vs. healthy:

fit1 <- aov(protwt~health*trip, data = prot.clad1.bl)

where protwt is protein per wet weight and all other factors are as defined above

Protein per wet weight for unbleached across three sites:

fit1 <- aov(protwt~site*trip, data = prot.clad.he)

where all factors are as defined above.

**Tables**

**Table S1.** Top: Abundance of octocorals across three reefs in the Lord Howe Island lagoon, ANOVA of GLMM, and pairwise comparisons. Pairwise comparisons are only reported against the group of interest, octocoral. Bottom: Percent cover of octocoral by site, ANOVA of GLMM results comparing percent cover across sites and trips, and pairwise comparisons. An interaction term was not included as the experimental design did not have statistical power to test for them. Both analyses were performed using a negative binomial distribution (nbinom1). Significant results are highlighted in bold.

| **Benthic group cover** | | | |
| --- | --- | --- | --- |
| Group | N | Mean | SE |
| Octocoral | 135 | 6.6% | 1.3% |
| Stony coral | 135 | 15.1% | 1.5% |
| algae | 135 | 38.8% | 2.4% |
| seagrass | 135 | 2.8% | 1.2% |
| Abiotic factors | 135 | 21.4% | 2.2%s |
| **ANOVA of GLMM of benthic group analysis** | | | |
|  | χ^2^ | Df | p-value |
| Benthic group | 231.12 | 4 | **<2.23-16** |
| **Pairwise comparisons – comparisons with octocoral only** | | | |
|  | t ratio | Df | p-value |
| Octocoral – abiotic | 6.65 | 666 | **<0.0001** |
| Octocoral – algae | 11.44 | 666 | **<0.0001** |
| Octocoral – seagrass | -5.20 | 666 | **<0.0001** |
| Octocoral – stony coral | -5.66 | 666 | **<0.0001** |

| **Octocoral cover** | | | |
| --- | --- | --- | --- |
| Site | N | Mean | SE |
| Sylphs Hole | 27 | 1.29% | 0.91% |
| North Bay | 27 | 0.37% | 0.26% |
| Coral Gardens | 27 | 17.22% | 4.15% |
| Comets Hole | 27 | 5.37% | 1.48% |
| Erscotts Reef | 27 | 8.52% | 2.37% |
| ANOVA **of GLMM across sites and trips of octocoral cover** | | | |
|  | χ^2^ | Df | p-value |
| Site | 24.10 | 4 | **7.64e-05** |
| Trip | 1.23 | 2 | 0.54 |
| **Pairwise comparisons** | | | |
|  | t ratio | Df | p-value |
| *Site* |  |  |  |
| Comets Hole – Coral Gardens | -2.04 | 126 | 0.25 |
| Comets Hole – Erscotts Reef | -0.71 | 126 | 0.95 |
| Comets Hole – North Bay | 2.45 | 126 | 0.11 |
| Comets Hole – Sylphs Hole | 2.35 | 126 | 0.14 |
| Coral Gardens – Erscotts Reef | 1.36 | 126 | 0.65 |
| Coral Gardens – North Bay | 3.72 | 126 | **0.003** |
| Coral Gardens – Sylphs Hole | 3.62 | 126 | **0.004** |
| Erscotts Reef – North Bay | 2.91 | 126 | **0.03** |
| Erscotts Reef – Sylph’ Hole | 2.81 | 126 | **0.04** |
| North Bay – Sylphs Hole | -0.08 | 126 | 0.99 |
| *Monitoring interval* |  |  |  |
| Mar – Apr/May | 1.10 | 126 | 0.52 |
| Mar – Oct | 3.36 | 126 | 0.93 |
| April/May - October | -0.78 | 126 | 0.72 |

**Table S2.** Abundance and species composition of octocorals and *Entacmaea quadricolor* anemones by site and monitoring interval, followed by species composition and abundance differences. GLMM results and pairwise comparisons are reported Top, and MANOVA results across all three sites and between pairs of sites with MDS stress are reported Bottom. If models contained significant interactions pairwise comparisons were not conducted on single factors. Distribution family for each comparison is as follows: octocorals – nbinom1; *Entacmaea quadricolor* – Poisson. Significant results are highlighted in bold.

| **ANOVA of GLMM by group** | | | | | | | | | |
| --- | --- | --- | --- | --- | --- | --- | --- | --- | --- |
|  | **Octocorals** | | | | | ***Entacmaea quadricolor*** | | | |
|  | χ^2^ | | Df | p-value | | χ^2^ | Df | | p-value |
| Site | 85.09 | | 2 | **<2e-16** | | 0.59 | 2 | | 0.74 |
| Monitoring interval | 0.52 | | 2 | 0.77 | | 24.99 | 2 | | **3.74e-06** |
| Site*Monitoring interval | 0.34 | | 4 | 0.99 | | 10.07 | 4 | | **0.04** |
| **Pairwise comparisons** | | | | | | | | | |
|  | t ratio | | Df | p-value | | t ratio | Df | | p-value |
| *Site* | | | | | | | | | |
| Coral Gardens – North Bay | 7.03 | | 124 | **<0.0001** | | **--** | -- | | -- |
| Coral Gardens – Sylph’s Hole | 7.10 | | 124 | **<0.0001** | | -- | -- | | -- |
| North Bay – Sylph’s Hole | 0.71 | | 124 | 0.99 | | -- | -- | | -- |
| *Monitoring interval* |  | |  |  | |  |  | |  |
| Mar – Apr/May | -0.45 | | 124 | 0.90 | | **--** | -- | | -- |
| Mar – Oct | 0.09 | | 124 | 0.99 | | -- | -- | | -- |
| Apr/May – Oct | 0.85 | | 124 | 0.85 | | -- | -- | | -- |
| *Monitoring interval by site* | | | | | | | | | |
| Coral Gardens |  | |  |  | |  |  | |  |
| Mar – Apr/May | -0.36 | | 124 | 0.93 | | 0.43 | 17 | | 0.91 |
| Mar – Oct | 0.24 | | 124 | 0.97 | | -3.57 | 17 | | **0.006** |
| Apr/May – Oct | 0.61 | | 124 | 0.82 | | -3.84 | 17 | | **0.004** |
| North Bay |  | |  |  | |  |  | |  |
| Mar – Apr/May | -0.16 | | 124 | 0.99 | | 0.34 | 17 | | 0.93 |
| Mar – Oct | 0.40 | | 124 | 0.91 | | -0.48 | 17 | | 0.88 |
| Apr/May – Oct | 0.55 | | 124 | 0.85 | | -0.82 | 17 | | .70 |
| Sylph’s Hole |  | |  |  | |  |  | |  |
| Mar – Apr/May | -0.35 | | 124 | 0.94 | | -0.88 | 17 | | 0.66 |
| Mar – Oct | -0.37 | | 124 | 0.93 | | -3.21 | 17 | | **0.01** |
| Apr/May – Oct | -0.02 | | 124 | 0.99 | | -2.37 | 17 | | 0.07 |
| *Site by monitoring interval* | | | | | | | | | |
| 1 |  | |  |  | |  |  | |  |
| Coral Gardens – North Bay | 4.16 | | 124 | **0.0002** | | -0.93 | 17 | | 0.63 |
| Coral Gardens – Sylph’s Hole | 4.12 | | 124 | **0.0002** | | -0.98 | 17 | | 0.60 |
| North Bay – Sylph’s Hole | 0.36 | | 124 | 0.93 | | -0.06 | 17 | | 0.99 |
| 2 |  | |  |  | |  |  | |  |
| Coral Gardens – North Bay | 4.19 | | 124 | **0.0002** | | -0.97 | 17 | | 0.61 |
| Coral Gardens – Sylph’s Hole | 4.39 | | 124 | **0.0001** | | -1.23 | 17 | | 0.45 |
| North Bay – Sylph’s Hole | 0.18 | | 124 | 0.98 | | -0.26 | 17 | | 0.96 |
| 3 |  | |  |  | |  |  | |  |
| Coral Gardens – North Bay | 4.04 | | 124 | **0.0003** | | -0.18 | 17 | | 0.98 |
| Coral Gardens – Sylph’s Hole | 4.01 | | 124 | **0.0003** | | -0.52 | 17 | | 0.86 |
| North Bay – Sylph’s Hole | -0.41 | | 124 | 0.91 | | -0.34 | 17 | | 0.93 |
| **PERMANOVA results of square-root transformed octocoral and anemone species composition by site** | | | | | | | | | |
| Full model. Stress = 0.13 | | | | | | | | | |
|  | SumSq | df | F | R2 | p(PERMANOVA) | | | p(Dispersion) | |
| Site | 3.24 | 2 | 14.08 | 0.599 | **0.001** | | | 0.117 | |
| Monitoring interval | 0.08 | 2 | 0.33 | 0.01 | 0.92 | | | 0.113 | |
| Site*Monitoring interval | 0.14 | 4 | 0.30 | 0.03 | 0.99 | | | --- | |
| Residuals | 1.95 | 17 |  | 0.11 |  | | |  | |
| Pairwise comparisons | SumSq | Df | F | R2 | p(PERMANOVA) adjusted | | |  | |
| North Bay vs. Sylphs Hole | 1.27 | 1 | 10.16 | 0.40 | **0.003** | | | -- | |
| North Bay vs. Coral Gardens | 1.76 | 1 | 21.33 | 0.57 | **0.003** | | | -- | |
| Sylphs Hole vs. Coral Gardens | 1.82 | 1 | 23.80 | 0.61 | **0.003** | | | -- | |

**Table S3.** Table of results for *Cladiella* sp. 1 comparing Symbiodiniaceae per µg protein, chlorophyll a per µg protein, chlorophyll c_2_ per µg protein, photosynthetic yield, chlorophyll a per Symbiodiniaceae, chlorophyll c_2_ per Symbiodiniaceae, and µg protein per g wet weight between healthy and bleached corals at Sylph’s Hole during monitoring intervals, peak bleaching and early recovery. If interactions were significant pairwise comparisons were not conducted on single factors. Distributions used in GLMM models were as follows: Symbiodiniaceae per µg protein – Poisson; all remaining – Gaussian. Significant results are highlighted in bold.

| Predictor | Symbiodiniaceae per µg protein | | | | | | | | Log(chlorophyll a per µg protein) | | | | | Log(chlorophyll c per µg protein) | | |
| --- | --- | --- | --- | --- | --- | --- | --- | --- | --- | --- | --- | --- | --- | --- | --- | --- |
| ANOVA **of GLMM** | | | | | | | | |  | | | | |  | | |
|  | χ^2^ | | Df | | | p-value | | | χ^2^ | | DF | | p-value | χ^2^ | DF | p-value |
| Monitoring interval | 1.25 | | 1 | | | 0.26 | | | 3.85 | | 1 | | **0.049** | 2.10 | 1 | 0.15 |
| Health | 135.31 | | 1 | | | **<2e-16** | | | 18.84 | | 1 | | **1.42e-05** | 123.07 | 1 | **<2e-16** |
| Monitoring interval*Health | 0.96 | | 1 | | | 0.33 | | | 0.003 | | 1 | | 0.096 | 0.02 | 1 | 0.89 |
| **Pairwise comparisons** | | | | | | | | | | | | | | | | |
|  | t ratio | | df | | | p-value | | | t ratio | | df | | p-value | t ratio | df | p-value |
| *Health* | | | | | | | | | | | | | | | | |
| Bl – He | -11.6 | | 241 | | | **<0.0001** | | | -4.33 | | 36 | | **0.0001** | -11.08 | 36 | **<0.0001** |
| *Monitoring interval* | | | | | | | | | | | | | | | | |
| March-April/May | -1.13 | | 241 | | | 0.26 | | | -1.96 | | 36 | | 0.06 | -1.44 | 36 | 0.16 |
| *Monitoring interval by health* | | | | | | | | | | | | | | | | |
| Bleached |  | |  | | |  | | |  | |  | |  |  |  |  |
| March-April/May | -1.48 | | 241 | | | 0.14 | | | -1.38 | | 36.2 | | 0.18 | -1.15 | 36.1 | 0.26 |
| Healthy |  | |  | | |  | | |  | |  | |  |  |  |  |
| March-April/May | -0.11 | | 241 | | | 0.92 | | | -1.39 | | 35.9 | | 0.17 | -0.90 | 36 | 0.38 |
| *Health by Monitoring interval* | | | | | | | | | | | | | | | | |
| March |  | |  | | |  | | |  | |  | |  |  |  |  |
| Bl – He | -8.97 | | 241 | | | **<0.0001** | | | -2.94 | | 36.5 | | **0.0057** | -7.74 | 36.2 | **<0.0001** |
| April/May |  | |  | | |  | | |  | |  | |  |  |  |  |
| Bl – He | -7.48 | | 241 | | | **<0.0001** | | | -3.194 | | 35.4 | | **0.003** | -7.95 | 35.9 | **<0.0001** |
| **ANOVA of GLMM (photosynthetic yield) or ANOVA (chlorophyll per Symbiodiniaceae)** | | | | | | | | | | | | | | | | |
| Predictor | Photosynthetic yield | | | | | | | | Log(chlorophyll a per Symbiodiniaceae) | | | | | Log(chlorophyll c_2_ per Symbiodiniaceae) | | |
|  |  | | | | | | | |  | |  | |  |  |  |  |
|  | χ^2^ | | DF | | | p-value | | | F-value | | DF | | p-value | F-value | DF | p-value |
| Monitoring interval | 8.55 | | 1 | | | **0.003** | | | 1.18 | | 1 | | 0.28 | 1.50 | 1 | 0.23 |
| Health | 27.72 | | 1 | | | **1.403-07** | | | 5.44 | | 1 | | **0.03** | 16.76 | 1 | **0.0002** |
| Monitoring interval*Health | 23.11 | | 1 | | | **1.53e-06** | | | 0.001 | | 1 | | 0.97 | 0.07 | 1 | 0.80 |
| Equivalence test – April/May | | | | | | | | | | | | | | | | |
| Equivalence level | p (5%) | | p (10%) | | | p (20%) | | | -- | | -- | | -- | -- | -- | -- |
| Bl - He | 0.23 | | **0.04** | | | **--** | | | -- | | -- | | -- | -- | -- | -- |
| **Pairwise comparisons** | | | | | | | | | | | | | | | | |
|  | t ratio | | df | | | p-value | | | t ratio | | df | | p-value | t ratio | df | p-value |
| *Health* | | | | | | | | | | | | | | | | |
| Bl – He | -- | | -- | | | -- | | | 2.33 | | 36 | | **0.03** | 4.10 | 36 | **0.0002** |
| *Monitoring interval* | | | | | | | | | | | | | | | | |
| March-April/May | -- | | -- | | | -- | | | -1.09 | | 36 | | 0.28 | -1.25 | 36 | 0.22 |
| *Monitoring interval by health* | | | | | | | | | | | | | | | | |
| Bleached |  | |  | | |  | | |  | |  | |  |  |  |  |
| March-April/May | -5.43 | | 36 | | | **<.0001** | | | -0.77 | | 36 | | 0.45 | -0.72 | 36 | 0.48 |
| Healthy |  | |  | | |  | | |  | |  | |  |  |  |  |
| March-April/May | 1.47 | | 36 | | | 0.15 | | | -0.78 | | 36 | | 0.44 | -1.04 | 36 | 0.31 |
| *Health by Monitoring interval* | | | | | | | | | | | | | | | | |
| March |  | |  | | |  | | |  | |  | |  |  |  |  |
| Bl – He | -7.12 | | 36 | | | **<.0001** | | | 1.63 | | 36 | | 0.11 | 3.01 | 36 | **0.005** |
| April/May |  | |  | | |  | | |  | |  | |  |  |  |  |
| Bl – He | -0.34 | | 36 | | | 0.73 | | | 1.66 | | 36 | | 0.10 | 2.79 | 36 | **0.008** |
| predictor | | Protein per wet weight | | | | | | | | | |  |  |  |  |  |
| **ANOVA** | |  | |  | |  |  | | |  | |  |  |  |  |  |
|  | | Sum sq | | Mean sq | | Df | F-value | | | p-value | |  |  |  |  |  |
| Health | | 1.3e6 | | 1.3e6 | | 1 | 7.72 | | | **0.008** | |  |  |  |  |  |
| Monitoring interval | | 1227 | | 1227 | | 1 | 0.007 | | | 0.93 | |  |  |  |  |  |
| Health*monitoring interval | | 2.6e6 | | 2.6e6 | | 1 | 14.92 | | | **0.0004** | |  |  |  |  |  |
| Residuals | | 6.4e6 | | 176391 | | 36 |  | | |  | |  |  |  |  |  |
| **Pairwise comparisons** | | | | | | | | | | | |  |  |  |  |  |
|  | | t ratio | | | df | | | p-value | | | |  |  |  |  |  |
| *Health* | | | | | | | | | | | |  |  |  |  |  |
| Bl – He | | -- | | | -- | | | -- | | | |  |  |  |  |  |
|  | | | | | | | | | | | |  |  |  |  |  |
| March-April/May | | -- | | | -- | | | -- | | | |  |  |  |  |  |
|  | | | | | | | | | | | |  |  |  |  |  |
| Bleached | |  | | |  | | |  | | | |  |  |  |  |  |
| March-April/May | | -2.79 | | | 36 | | | **0.008** | | | |  |  |  |  |  |
| Healthy | |  | | |  | | |  | | | |  |  |  |  |  |
| March-April/May | | 2.67 | | | 36 | | | **0.01** | | | |  |  |  |  |  |
|  | | | | | | | | | | | |  |  |  |  |  |
| March | |  | | |  | | |  | | | |  |  |  |  |  |
| Bl – He | | -4.70 | | | 36 | | | **<0.0001** | | | |  |  |  |  |  |
| April/May | |  | | |  | | |  | | | |  |  |  |  |  |
| Bl – He | | 0.77 | | | 36 | | | 0.45 | | | |  |  |  |  |  |
| Non-inferiority test – April/May | | | | | | | | | | | |  |  |  |  |  |
| Equivalence level | | p (5%) | | | p (10%) | | | p (20%) | | | |  |  |  |  |  |
|  | | 0.22 | | | 0.22 | | | 0.22 | | | |  |  |  |  |  |

**Table S4.** Table of results for *Cladiella* sp. 1 comparing Symbiodiniaceae per µg protein, chlorophyll a and chlorophyll c_2_ per µg protein, chlorophyll a and chlorophyll c_2_ per Symbiodiniaceae cell, and µg of protein per gram of wet weight. Non-significant interactions and factors not involved in interactions were removed from models. If models contained interactions pairwise comparisons were not conducted on single factors as results could be misleading. Samples were not able to be collected at North Bay during monitoring interval 1, comparisons with this are indicated as dashed lines. Distributions used in GLMM models were as follows: Symbiodiniaceae – Poisson, all other factors – Gaussian. Significant results are highlighted in bold.

| Predictor | | Symbiodiniaceae per µg protein | | | | | | | | | | | Log(chlorophyll a per µg protein) | | | | | | | | | Log(chlorophyll c per µg protein) | | | | | | |
| --- | --- | --- | --- | --- | --- | --- | --- | --- | --- | --- | --- | --- | --- | --- | --- | --- | --- | --- | --- | --- | --- | --- | --- | --- | --- | --- | --- | --- |
| **ANOVA of GLMM** | | | | | | | | | | | | |  | | | | | | | | |  | | | | | | |
|  | | χ^2^ | | | Df | | | | p-value | | | | χ^2^ | | | DF | | p-value | | | | χ^2^ | | DF | | | p-value | |
| Monitoring interval | | **20.53** | | | **3** | | | | **0.0001** | | | | **25.52** | | | **2** | | **2.88e-06** | | | | **21.12** | | **2** | | | **2.60e-05** | |
| Site | | 4.77 | | | 3 | | | | 0.19 | | | | **23.33** | | | **2** | | **8.60e-06** | | | | **25.28** | | **2** | | | **3.25e-06** | |
| Monitoring interval*Site | | **14.82** | | | **4** | | | | **0.005** | | | | **40.31** | | | **3** | | **9.17e-09** | | | | **40.75** | | **3** | | | **7.38e-09** | |
| **Pairwise comparisons** | | | | | | | | | | | | |  | | |  | |  | | | |  | |  | | |  | |
|  | | t ratio | | | df | | | | p-value | | | | t ratio | | | df | | p-value | | | | t ratio | | df | | | p-value | |
| *Site only* | | | | | | | | | | | | |  | | |  | |  | | | |  | |  | | |  | |
| CG-NB | | -- | | | -- | | | | -- | | | | -- | | | -- | | -- | | | | -- | | -- | | | -- | |
| CG-Syl | | -- | | | -- | | | | -- | | | | -- | | | -- | | -- | | | | -- | | -- | | | -- | |
| NB-Syl | | -- | | | -- | | | | -- | | | | -- | | | -- | | -- | | | | -- | | -- | | | -- | |
| *Monitoring interval only* | | | | | | | | | | | | |  | | |  | |  | | | |  | |  | | |  | |
| March-April/May | | -- | | | -- | | | | -- | | | | -- | | | -- | | -- | | | | -- | | -- | | | -- | |
| March-October | | -- | | | -- | | | | -- | | | | -- | | | -- | | -- | | | | -- | | -- | | | -- | |
| April/May-October | | -- | | | -- | | | | -- | | | | -- | | | -- | | -- | | | | -- | | -- | | | -- | |
| *Monitoring interval by site* | | | | | | | | | | | | |  | | |  | |  | | | |  | |  | | |  | |
| Coral Gar. | |  | | |  | | | |  | | | |  | | |  | |  | | | |  | |  | | |  | |
| March-April/May | | -0.47 | | | 458 | | | | 0.89 | | | | -0.04 | | | 69.0 | | 0.99 | | | | -1.91 | | 69.0 | | | 0.14 | |
| March-October | | -0.83 | | | 458 | | | | 0.69 | | | | 0.15 | | | 69.0 | | 0.99 | | | | -0.12 | | 69.0 | | | 0.99 | |
| April/May-October | | 0.38 | | | 458 | | | | 0.92 | | | | 0.18 | | | 68.9 | | 0.98 | | | | 1.79 | | 68.1 | | | 0.19 | |
| North Bay | |  | | |  | | | |  | | | |  | | |  | |  | | | |  | |  | | |  | |
| March-April/May | | -- | | | -- | | | | -- | | | | -- | | | -- | | -- | | | | -- | | -- | | | -- | |
| March-October | | -- | | | -- | | | | -- | | | | -- | | | -- | | -- | | | | -- | | -- | | | -- | |
| April/May-October | | 0.33 | | | 458 | | | | 0.94 | | | | 0.022 | | | 69.0 | | 0.99 | | | | 3.57 | | 68.9 | | | **0.0019** | |
| Sylph’s | |  | | |  | | | |  | | | |  | | |  | |  | | | |  | |  | | |  | |
| March-April/May | | -0.14 | | | 458 | | | | 0.99 | | | | -1.22 | | | 69.0 | | 0.45 | | | | -2.56 | | 69.0 | | | **0.034** | |
| March-October | | -4.87 | | | 458 | | | | **<0.0001** | | | | -7.46 | | | 69.1 | | **<0.0001** | | | | -6.58 | | 69.8 | | | **<0.0001** | |
| April/May-October | | -4.75 | | | 458 | | | | **<0.0001** | | | | -6.42 | | | 69.0 | | **<0.0001** | | | | -4.16 | | 68.9 | | | **0.0003** | |
| *Site by Monitoring interval* | | | | | | | | | | | | |  | | |  | |  | | | |  | |  | | |  | |
| March | | | | | | | | | | | | |  | | |  | |  | | | |  | |  | | |  | |
| CG-NB | | -- | | | -- | | | | -- | | | | -- | | | -- | | -- | | | | -- | | -- | | | -- | |
| CG-Syl | | 1.80 | | | 458 | | | | 0.17 | | | | 5.39 | | | 69.1 | | **<0.0001** | | | | 4.93 | | 69.9 | | | **<0.0001** | |
| NB-Syl | | -- | | | -- | | | | -- | | | | -- | | | -- | | -- | | | | -- | | -- | | | -- | |
| April/May | | | | | | | | | | | | |  | | |  | |  | | | |  | |  | | |  | |
| CG-NB | | -0.10 | | | 458 | | | | 0.99 | | | | -0.50 | | | 69.0 | | 0.87 | | | | -1.31 | | 68.9 | | | 0.39 | |
| CG-Syl | | 2.21 | | | 458 | | | | 0.07 | | | | 4.47 | | | 68.9 | | **0.0001** | | | | 4.57 | | 68.1 | | | **0.0001** | |
| NB-Syl | | 2.30 | | | 458 | | | | 0.056 | | | | 4.97 | | | 69.0 | | **<0.0001** | | | | 5.86 | | 68.9 | | | **<0.0001** | |
| October | | | | | | | | | | | | |  | | |  | |  | | | |  | |  | | |  | |
| CG-NB | | -0.04 | | | 458 | | | | 0.99 | | | | -0.07 | | | 68.9 | | 0.79 | | | | 0.41 | | 68.1 | | | 0.91 | |
| CG-Syl | | -2.10 | | | 458 | | | | 0.09 | | | | -2.08 | | | 69.0 | | 0.10 | | | | -0.41 | | 68.9 | | | 0.34 | |
| NB-Syl | | -2.12 | | | 458 | | | | 0.09 | | | | -1.47 | | | 69.0 | | 0.31 | | | | -1.86 | | 68.9 | | | 0.16 | |
| Non-inferiority test – October | | | | | | | | | | | | | | | | | | | | | | | | | | | | |
| Equivalence level | | p (5%) | | | p (10%) | | | | p (20%) | | | | p (5%) | | | p (10%) | | p (20%) | | | | p (5%) | | p (10%) | | | p (20%) | |
| CG-NB | | 0.75 | | | -- | | | | -- | | | | 0.44 | | | 0.31 | | 0.14 | | | | 0.91 | | 0.85 | | | 0.64 | |
| CG-Syl | | **0.02** | | | -- | | | | **--** | | | | **<0.03** | | | **0.02** | | **0.004** | | | | 0.14 | | 0.09 | | | **0.03** | |
| NB-Syl | | **0.02** | | | -- | | | | **--** | | | | 0.12 | | | 0.07 | | **0.02** | | | | 0.055 | | **0.03** | | | **0.009** | |
| Predictor | Log(chlorophyll a per Symbiodiniaceae cell) | | | | | | | | | | | Log(chlorophyll c_2_ per Symbiodiniaceae cell) | | | | | | | | Protein per wet weight | | | | | | | | |
| **ANOVA** | | | | | | | | | | | | | | | | | | | | | | | | | | | | |
|  | Sum sq | | | Mean sq | | Df | F value | | | P value | | Sum sq | | Mean sq | | Df | F value | | P value | Sum sq | | | Mean sq | | Df | F value | | P value |
| Monitoring interval | 0.31 | | | 0.16 | | 2 | 5.23 | | | **0.008** | | 3.36 | | 1.68 | | 2 | 16.17 | | **1.7e-06** | 3.1e6 | | | 1.6e6 | | 2 | 6.78 | | **0.002** |
| Site | 1.83 | | | 0.92 | | 2 | 30.52 | | | **3.2e-10** | | 2.61 | | 1.30 | | 2 | 12.55 | | **2.2e-05** | 2.5e5 | | | 1.2e5 | | 2 | 0.53 | | 0.59 |
| Monitoring interval *Site | 0.99 | | | 0.33 | | 3 | 11.03 | | | **5.3e-06** | | 1.60 | | 0.53 | | 3 | 5.14 | | **0.003** | 6.2e6 | | | 2.1e6 | | 3 | 8.91 | | **0.00005** |
| Residuals | 2.08 | | | 0.03 | | 69 |  | | |  | | 7.16 | | 0.10 | | 69 |  | |  | 1.6e7 | | | 2.3e5 | | 68 |  | |  |
| **Pairwise comparisons** | | | | | | | | | | |  | | | |  | | | |  | |  | | |  | | | |  |
|  | | t ratio | | | df | | | | p-value | | t ratio | | | | df | | | | p-value | | t ratio | | | df | | | | p-value |
| *Site only* | | | | | | | | | | |  | | | |  | | | |  | |  | | |  | | | |  |
| CG-NB | | -- | | | -- | | | | -- | | -- | | | | -- | | | | -- | | -- | | | -- | | | | -- |
| CG-Syl | | -- | | | -- | | | | -- | | -- | | | | -- | | | | -- | | -- | | | -- | | | | -- |
| NB-Syl | | -- | | | -- | | | | -- | | -- | | | | -- | | | | -- | | -- | | | -- | | | | -- |
| *Monitoring interval only* | | | | | | | | | | |  | | | |  | | | |  | |  | | |  | | | |  |
| March-April/May | | -- | | | -- | | | | -- | | -- | | | | -- | | | | -- | | -- | | | -- | | | | -- |
| March-October | | -- | | | -- | | | | -- | | -- | | | | -- | | | | -- | | -- | | | -- | | | | -- |
| April/May-October | | -- | | | -- | | | | -- | | -- | | | | -- | | | | -- | | -- | | | -- | | | | -- |
| *Monitoring interval by site* | | | | | | | | | | |  | | | |  | | | |  | |  | | |  | | | |  |
| Coral Gar. | |  | | |  | | | |  | |  | | | |  | | | |  | |  | | |  | | | |  |
| March-April/May | | 0.73 | | | 69 | | | | 0.75 | | -2.04 | | | | 69 | | | | 0.11 | | -3.13 | | | 68 | | | | **0.007** |
| March-October | | 1.98 | | | 69 | | | | 0.12 | | 0.60 | | | | 69 | | | | 0.82 | | -3.28 | | | 68 | | | | **0.005** |
| April/May-October | | 1.30 | | | 69 | | | | 0.40 | | 2.65 | | | | 69 | | | | **0.03** | | -0.24 | | | 68 | | | | 0.97 |
| North Bay | |  | | |  | | | |  | |  | | | |  | | | |  | |  | | |  | | | |  |
| March-April/May | | -- | | | -- | | | | -- | | -- | | | | -- | | | | -- | | -- | | | -- | | | | -- |
| March-October | | -- | | | -- | | | | -- | | -- | | | | -- | | | | -- | | -- | | | -- | | | | -- |
| April/May-October | | 0.06 | | | 69 | | | | 0.71 | | 5.02 | | | | 69 | | | | **<0.0001** | | -2.18 | | | 68 | | | | 0.08 |
| Sylph’s | |  | | |  | | | |  | |  | | | |  | | | |  | |  | | |  | | | |  |
| March-April/May | | -1.26 | | | 69 | | | | 0.42 | | -2.51 | | | | 69 | | | | **0.04** | | 2.33 | | | 68 | | | | 0.06 |
| March-October | | -5.42 | | | 69 | | | | **<0.0001** | | -2.62 | | | | 69 | | | | **0.03** | | 2.90 | | | 68 | | | | **0.01** |
| April/May-October | | -4.28 | | | 69 | | | | **0.0002** | | -0.11 | | | | 69 | | | | 0.99 | | 0.57 | | | 68 | | | | 0.83 |
| *Site by Monitoring interval* | | | | | | | | | | |  | | | |  | | | |  | |  | | |  | | | |  |
| March | | | | | | | | | | |  | | | |  | | | |  | |  | | |  | | | |  |
| CG-NB | | -- | | | -- | | | | -- | | -- | | | | -- | | | | -- | | -- | | | -- | | | | -- |
| CG-Syl | | 6.95 | | | 69 | | | | **<0.0001** | | 3.72 | | | | 69 | | | | **0.001** | | -5.63 | | | 68 | | | | **>0.0001** |
| NB-Syl | | -- | | | -- | | | | -- | | -- | | | | -- | | | | -- | | -- | | | -- | | | | -- |
| April/May | | | | | | | | | | |  | | | |  | | | |  | |  | | |  | | | |  |
| CG-NB | | -0.83 | | | 69 | | | | 0.69 | | -1.55 | | | | 69 | | | | 0.27 | | 2.32 | | | 68 | | | | 0.99 |
| CG-Syl | | 5.28 | | | 69 | | | | **<0.0001** | | 3.44 | | | | 69 | | | | **0.003** | | -0.24 | | | 68 | | | | 0.79 |
| NB-Syl | | 6.11 | | | 69 | | | | **<0.0001** | | 4.99 | | | | 69 | | | | **<0.001** | | -2.56 | | | 68 | | | | **0.02** |
| October | | | | | | | | | | |  | | | |  | | | |  | |  | | |  | | | |  |
| CG-NB | | -1.35 | | | 69 | | | | 0.37 | | 0.72 | | | | 69 | | | | 0.75 | | 0.23 | | | 68 | | | | 0.93 |
| CG-Syl | | -0.33 | | | 69 | | | | 0.94 | | 0.59 | | | | 69 | | | | 0.83 | | 0.56 | | | 68 | | | | 0.97 |
| NB-Syl | | 1.05 | | | 69 | | | | 0.55 | | -0.14 | | | | 69 | | | | 0.99 | | 0.30 | | | 68 | | | | 0.94 |
| Non-inferiority test – October | | | | | | | | | | | | | | | | | | | | | | | | | | | | |
| equivalence level | | p (5%) | | | p (10%) | | | | p (20%) | | p (5%) | | | | p (10%) | | | | p (20%) | | p (5%) | | | p (10%) | | | | p (20%) |
| CG-NB | | 0.08 | | | **0.02** | | | | **0.008** | | 0.96 | | | | 0.89 | | | | 0.67 | | -- | | | -- | | | | -- |
| CG-Syl | | 0.43 | | | 0.19 | | | | **0.02** | | 0.94 | | | | 0.86 | | | | 0.59 | | -- | | | -- | | | | -- |
| NB-Syl | | 0.96 | | | 0.81 | | | | 0.26 | | 0.68 | | | | 0.51 | | | | 0.23 | | -- | | | -- | | | | -- |
| Predictor | | | Photosynthetic yield | | | | | | | |  |  |  |  |  |  |  |  |  |  |  |  |  |  |  |  |  |  |
| ANOVA **without interaction (for main effects)** | | | | | | | | | | |  |  |  |  |  |  |  |  |  |  |  |  |  |  |  |  |  |  |
|  | | | χ^2^ | | Df | | | p-value | | |  |  |  |  |  |  |  |  |  |  |  |  |  |  |  |  |  |  |
| Monitoring interval | | | 53.39 | | 2 | | | **2.55e-12** | | |  |  |  |  |  |  |  |  |  |  |  |  |  |  |  |  |  |  |
| Site | | | 10.15 | | 2 | | | **0.006** | | |  |  |  |  |  |  |  |  |  |  |  |  |  |  |  |  |  |  |
| ANOVA **with interaction (for equivalence testing)** | | | | | | | | | | |  |  |  |  |  |  |  |  |  |  |  |  |  |  |  |  |  |  |
| Monitoring interval | | | 53.12 | | 2 | | | **2.90e-12** | | |  |  |  |  |  |  |  |  |  |  |  |  |  |  |  |  |  |  |
| Site | | | 10.10 | | 2 | | | **0.006** | | |  |  |  |  |  |  |  |  |  |  |  |  |  |  |  |  |  |  |
| Monitoring interval*site | | | 2.65 | | 3 | | | 0.45 | | |  |  |  |  |  |  |  |  |  |  |  |  |  |  |  |  |  |  |
| **Pairwise comparisons** | | | | | | | | | | |  |  |  |  |  |  |  |  |  |  |  |  |  |  |  |  |  |  |
|  | | | t ratio | | df | | | p-value | | |  |  |  |  |  |  |  |  |  |  |  |  |  |  |  |  |  |  |
| *Site only – from non-interaction*  *ANOVA* | | | | | | | | | | |  |  |  |  |  |  |  |  |  |  |  |  |  |  |  |  |  |  |
| CG-NB | | | -1.15 | | 73 | | | 0.48 | | |  |  |  |  |  |  |  |  |  |  |  |  |  |  |  |  |  |  |
| CG-Syl | | | 2.15 | | 73 | | | 0.09 | | |  |  |  |  |  |  |  |  |  |  |  |  |  |  |  |  |  |  |
| NB-Syl | | | 3.04 | | 73 | | | **0.009** | | |  |  |  |  |  |  |  |  |  |  |  |  |  |  |  |  |  |  |
| *Monitoring interval only – from non-*  *interaction ANOVA* | | | | | | | | | | |  |  |  |  |  |  |  |  |  |  |  |  |  |  |  |  |  |  |
| March-April/May | | | 4.02 | | 73 | | | **0.0004** | | |  |  |  |  |  |  |  |  |  |  |  |  |  |  |  |  |  |  |
| March-October | | | 7.27 | | 73 | | | **<0.0001** | | |  |  |  |  |  |  |  |  |  |  |  |  |  |  |  |  |  |  |
| April/May-October | | | 3.91 | | 73 | | | **0.0006** | | |  |  |  |  |  |  |  |  |  |  |  |  |  |  |  |  |  |  |
| *Monitoring interval by site* | | | | | | | | | | |  |  |  |  |  |  |  |  |  |  |  |  |  |  |  |  |  |  |
| Coral Gar. | | |  | |  | | |  | | |  |  |  |  |  |  |  |  |  |  |  |  |  |  |  |  |  |  |
| March-April/May | | | 2.50 | | 70 | | | **0.03** | | |  |  |  |  |  |  |  |  |  |  |  |  |  |  |  |  |  |  |
| March-October | | | 5.81 | | 70 | | | **<0.0001** | | |  |  |  |  |  |  |  |  |  |  |  |  |  |  |  |  |  |  |
| April/May-October | | | 3.38 | | 70 | | | **0.0034** | | |  |  |  |  |  |  |  |  |  |  |  |  |  |  |  |  |  |  |
| North Bay | | | | | | | | | | |  |  |  |  |  |  |  |  |  |  |  |  |  |  |  |  |  |  |
| March-April/May | | | -- | | -- | | | -- | | |  |  |  |  |  |  |  |  |  |  |  |  |  |  |  |  |  |  |
| March-October | | | -- | | -- | | | -- | | |  |  |  |  |  |  |  |  |  |  |  |  |  |  |  |  |  |  |
| April/May-October | | | 2.15 | | 70 | | | 0.09 | | |  |  |  |  |  |  |  |  |  |  |  |  |  |  |  |  |  |  |
| Sylph’s | | | | | | | | | | |  |  |  |  |  |  |  |  |  |  |  |  |  |  |  |  |  |  |
| March-April/May | | | 2.84 | | 70 | | | **0.02** | | |  |  |  |  |  |  |  |  |  |  |  |  |  |  |  |  |  |  |
| March-October | | | 4.07 | | 70 | | | **0.0003** | | |  |  |  |  |  |  |  |  |  |  |  |  |  |  |  |  |  |  |
| April/May-October | | | 1.27 | | 70 | | | 0.42 | | |  |  |  |  |  |  |  |  |  |  |  |  |  |  |  |  |  |  |
| *Site by Monitoring interval* | | | | | | | | | | |  |  |  |  |  |  |  |  |  |  |  |  |  |  |  |  |  |  |
| Peak bleaching | | | | | | | | | | |  |  |  |  |  |  |  |  |  |  |  |  |  |  |  |  |  |  |
| CG-NB | | | -- | | -- | | | -- | | |  |  |  |  |  |  |  |  |  |  |  |  |  |  |  |  |  |  |
| CG-Syl | | | 1.66 | | 70 | | | 0.23 | | |  |  |  |  |  |  |  |  |  |  |  |  |  |  |  |  |  |  |
| NB-Syl | | | -- | | -- | | | -- | | |  |  |  |  |  |  |  |  |  |  |  |  |  |  |  |  |  |  |
| April/May | | | | | | | | | | |  |  |  |  |  |  |  |  |  |  |  |  |  |  |  |  |  |  |
| CG-NB | | | -0.27 | | 70 | | | 0.96 | | |  |  |  |  |  |  |  |  |  |  |  |  |  |  |  |  |  |  |
| CG-Syl | | | 2.12 | | 70 | | | 0.09 | | |  |  |  |  |  |  |  |  |  |  |  |  |  |  |  |  |  |  |
| NB-Syl | | | 2.39 | | 70 | | | 0.051 | | |  |  |  |  |  |  |  |  |  |  |  |  |  |  |  |  |  |  |
| October | | | | | | | | | | |  |  |  |  |  |  |  |  |  |  |  |  |  |  |  |  |  |  |
| CG-NB | | | -1.55 | | 70 | | | 0.27 | | |  |  |  |  |  |  |  |  |  |  |  |  |  |  |  |  |  |  |
| CG-Syl | | | -0.08 | | 70 | | | 0.99 | | |  |  |  |  |  |  |  |  |  |  |  |  |  |  |  |  |  |  |
| NB-Syl | | | 1.51 | | 70 | | | 0.29 | | |  |  |  |  |  |  |  |  |  |  |  |  |  |  |  |  |  |  |
| Non-inferiority test – March | | | | | | | | | | |  |  |  |  |  |  |  |  |  |  |  |  |  |  |  |  |  |  |
| Equivalence level | | | p (5%) | | p (10%) | | | p (20%) | | |  |  |  |  |  |  |  |  |  |  |  |  |  |  |  |  |  |  |
| CG-NB | | | -- | | -- | | | -- | | |  |  |  |  |  |  |  |  |  |  |  |  |  |  |  |  |  |  |
| CG-Syl | | | 0.73 | | **0.03** | | | **<0.0001** | | |  |  |  |  |  |  |  |  |  |  |  |  |  |  |  |  |  |  |
| NB-Syl | | | -- | | -- | | | -- | | |  |  |  |  |  |  |  |  |  |  |  |  |  |  |  |  |  |  |
| Non-inferiority test – April/May | | | | | | | | | | |  |  |  |  |  |  |  |  |  |  |  |  |  |  |  |  |  |  |
| Equivalence level | | | p (5%) | | p (10%) | | | p (20%) | | |  |  |  |  |  |  |  |  |  |  |  |  |  |  |  |  |  |  |
| CG-NB | | | 0.11 | | **0.0005** | | | **<0.0001** | | |  |  |  |  |  |  |  |  |  |  |  |  |  |  |  |  |  |  |
| CG-Syl | | | 0.88 | | 0.08 | | | **<0.0001** | | |  |  |  |  |  |  |  |  |  |  |  |  |  |  |  |  |  |  |
| NB-Syl | | | 0.94 | | 0.14 | | | **<0.0001** | | |  |  |  |  |  |  |  |  |  |  |  |  |  |  |  |  |  |  |
| Non-inferiority test – October | | | | | | | | | | |  |  |  |  |  |  |  |  |  |  |  |  |  |  |  |  |  |  |
| Equivalence level | | | p (5%) | | p (10%) | | | p (20%) | | |  |  |  |  |  |  |  |  |  |  |  |  |  |  |  |  |  |  |
| CG-NB | | | 0.67 | | **0.03** | | | **<0.0001** | | |  |  |  |  |  |  |  |  |  |  |  |  |  |  |  |  |  |  |
| CG-Syl | | | 0.08 | | **0.0003** | | | **<0.0001** | | |  |  |  |  |  |  |  |  |  |  |  |  |  |  |  |  |  |  |
| NB-Syl | | | 0.63 | | **0.02** | | | **<0.0001** | | |  |  |  |  |  |  |  |  |  |  |  |  |  |  |  |  |  |  |

**Table S5.** Table of results for *Xenia* cf *crassa* comparing Symbiodiniaceae per µg protein, chlorophyll a and chlorophyll c_2_ per µg protein, and chlorophyll a and chlorophyll c_2_ per Symbiodiniaceae cell. Non-significant interactions and factors not involved in interactions were removed from models. If models contained significant interactions pairwise comparisons were not conducted on single factors. Significant values are indicated in bold. Distributions used in GLMM models were as follows: Symbiodiniaceae – Poisson, all other factors – Gaussian. Significant results are highlighted in bold.

| Predictor | | Symbiodiniaceae per µg protein | | | | | | | | | | | Log(chlorophyll a per µg protein) | | | | | | | | Log(chlorophyll c_2_ per µg protein) | | |
| --- | --- | --- | --- | --- | --- | --- | --- | --- | --- | --- | --- | --- | --- | --- | --- | --- | --- | --- | --- | --- | --- | --- | --- |
| ANOVA **of GLMM** | | | | | | | | | | | | |  | | | | | | | |  | | |
|  | | χ^2^ | | | Df | | | | p-value | | | | χ^2^ | | | DF | | | p-value | | χ^2^ | DF | p-value |
| Trip | | 25.40 | | | 2 | | | | **3.05e-06** | | | | 26.49 | | | 2 | | | **1.77e-06** | | 18.37 | 2 | **0.0001** |
| Site | | 2.25 | | | 2 | | | | 0.32 | | | | 0.66 | | | 2 | | | 0.72 | | 6.099 | 2 | **0.047** |
| Trip*Site | | 5.71 | | | 4 | | | | 0.22 | | | | 12.53 | | | 4 | | | **0.01** | | 10.58 | 4 | **0.03** |
| **Pairwise comparisons** | | | | | | | | | | | | |  | | |  | | |  | |  |  |  |
|  | | t ratio | | | df | | | | p-value | | | | t ratio | | | df | | | p-value | | t ratio | df | p-value |
| *Site only* | | | | | | | | | | | | |  | | |  | | |  | |  |  |  |
| CG-NB | | -0.73 | | | 517 | | | | 0.74 | | | | -- | | | -- | | | -- | | -- | -- | -- |
| CG-Syl | | 0.74 | | | 517 | | | | 0.74 | | | | -- | | | -- | | | -- | | -- | -- | -- |
| NB-Syl | | 1.48 | | | 517 | | | | 0.30 | | | | -- | | | -- | | | -- | | -- | -- | -- |
| *Trip only* | | | | | | | | | | | | |  | | |  | | |  | |  |  |  |
| Mar – Apr/May | | 5.07 | | | 517 | | | | **<0.0001** | | | | -- | | | -- | | | -- | | -- | -- | -- |
| Mar – Oct | | 2.85 | | | 517 | | | | **0.013** | | | | -- | | | -- | | | -- | | -- | -- | -- |
| Apr/May – Oct | | -2.29 | | | 517 | | | | 0.06 | | | | -- | | | -- | | | -- | | -- | -- | -- |
| *Trip by site* | | | | | | | | | | | | |  | | |  | | |  | |  |  |  |
| Coral Gar. | |  | | |  | | | |  | | | |  | | |  | | |  | |  |  |  |
| Mar – Apr/May | | 3.79 | | | 517 | | | | **0.0005** | | | | 2.47 | | | 78.7 | | | **0.04** | | 0.32 | 78.3 | 0.94 |
| Mar – Oct | | 0.84 | | | 517 | | | | 0.68 | | | | 0.88 | | | 86.0 | | | 0.65 | | -0.10 | 91.2 | 0.99 |
| Apr/May – Oct | | -2.99 | | | 517 | | | | **0.008** | | | | -1.88 | | | 85.5 | | | 0.15 | | -0.36 | 90.4 | 0.93 |
| North Bay | |  | | |  | | | |  | | | |  | | |  | | |  | |  |  |  |
| Mar – Apr/May | | 2.52 | | | 517 | | | | **0.03** | | | | 2.24 | | | 78.7 | | | 0.07 | | 1.41 | 78.3 | 0.34 |
| Mar – Oct | | 0.37 | | | 517 | | | | 0.36 | | | | 2.43 | | | 78.7 | | | **0.05** | | 2.09 | 78.3 | 0.099 |
| Apr/May – Oct | | -1.19 | | | 517 | | | | 0.46 | | | | 0.19 | | | 78.7 | | | 0.98 | | 0.69 | 78.3 | 0.77 |
| Sylph’s | |  | | |  | | | |  | | | |  | | |  | | |  | |  |  |  |
| Mar – Apr/May | | 2.46 | | | 517 | | | | **0.04** | | | | 1.73 | | | 86.0 | | | 0.19 | | 2.18 | 91.2 | 0.08 |
| Mar – Oct | | 2.73 | | | 517 | | | | **0.02** | | | | 4.94 | | | 86.2 | | | **<0.0001** | | 4.86 | 91.9 | **<0.0001** |
| Apr/May – Oct | | 0.27 | | | 517 | | | | 0.96 | | | | 2.88 | | | 78.9 | | | **0.01** | | 2.52 | 78.8 | **0.03** |
| *Site by Trip* | | | | | | | | | | | | |  | | |  | | |  | |  |  |  |
| March | |  | | |  | | | |  | | | |  | | |  | | |  | |  |  |  |
| CG-NB | | -0.17 | | | 517 | | | | 0.98 | | | | -0.49 | | | 78.7 | | | 0.87 | | -0.60 | 78.3 | 0.81 |
| CG-Syl | | 0.29 | | | 517 | | | | 0.95 | | | | -0.98 | | | 86.0 | | | 0.59 | | -3.20 | 91.2 | **0.005** |
| NB-Syl | | 0.46 | | | 517 | | | | 0.89 | | | | -0.40 | | | 85.5 | | | 0.92 | | -2.46 | 90.4 | **0.04** |
| April/May | |  | | |  | | | |  | | | |  | | |  | | |  | |  |  |  |
| CG-NB | | -1.46 | | | 517 | | | | 0.31 | | | | -0.07 | | | 78.7 | | | 0.75 | | 0.48 | 78.3 | 0.88 |
| CG-Syl | | -1.14 | | | 517 | | | | 0.49 | | | | -1.82 | | | 78.7 | | | 0.17 | | -1.26 | 78.3 | 0.42 |
| NB-Syl | | 0.33 | | | 517 | | | | 0.94 | | | | -1.12 | | | 78.7 | | | 0.27 | | -1.79 | 78.3 | 0.18 |
| October | |  | | |  | | | |  | | | |  | | |  | | |  | |  |  |  |
| CG-NB | | 0.39 | | | 517 | | | | 0.92 | | | | 1.34 | | | 86.0 | | | 0.38 | | 1.64 | 91.2 | 0.23 |
| CG-Syl | | 2.19 | | | 517 | | | | 0.07 | | | | 3.08 | | | 86.2 | | | **0.008** | | 1.68 | 91.9 | 0.21 |
| NB-Syl | | 1.80 | | | 517 | | | | 0.17 | | | | 1.57 | | | 78.9 | | | 0.27 | | 0.04 | 78.8 | 0.99 |
| Predictor | Log(chlorophyll a per Symbiodiniaceae cell) | | | | | | | | | | | Log(chlorophyll c_2_ per Symbiodiniaceae cell) | | | | | | | | |  |  |  |
| ANOVA | | | | | | | | | | | |  | | | | | | | | |  |  |  |
|  | Sum sq | | Mean sq | Df | | | F value | | | P value | | Sum sq | | Mean sq | | Df | F value | | | P value |  |  |  |
| Trip | 2.95 | | 1.48 | 2 | | | 16.93 | | | **7.59e-07** | | 5.20 | | 2.60 | | 2 | 9.60 | | | **0.0002** |  |  |  |
| Site | 0.37 | | 0.19 | 2 | | | 2.14 | | | 0.12 | | 1.92 | | 0.96 | | 2 | 3.55 | | | **0.03** |  |  |  |
| Trip*Site | 1.57 | | 0.37 | 4 | | | 4.20 | | | **0.004** | | 3.63 | | 0.91 | | 4 | 3.35 | | | **0.01** |  |  |  |
| Residuals | 6.89 | | 0.09 | 79 | | |  | | |  | | 21.39 | | 0.27 | | 79 |  | | |  |  |  |  |
| **Pairwise comparisons** | | | | | | | | | | |  | | | |  | | |  | | |  |  |  |
|  | t ratio | | | | | df | | p-value | | | t ratio | | | | df | | | p-value | | |  |  |  |
| *Site only* | | | | | | | | | | |  | | | |  | | |  | | |  |  |  |
| CG-NB | -- | | | | | -- | | -- | | | -- | | | | -- | | | -- | | |  |  |  |
| CG-Syl | -- | | | | | -- | | -- | | | -- | | | | -- | | | -- | | |  |  |  |
| NB-Syl | -- | | | | | -- | | -- | | | -- | | | | -- | | | -- | | |  |  |  |
| *Trip only* | | | | | | | | | | |  | | | |  | | |  | | |  |  |  |
| Mar – Apr/May | -- | | | | | -- | | -- | | | -- | | | | -- | | | -- | | |  |  |  |
| Mar – Oct | -- | | | | | -- | | -- | | | -- | | | | -- | | | -- | | |  |  |  |
| Apr/May – Oct | -- | | | | | -- | | -- | | | -- | | | | -- | | | -- | | |  |  |  |
| *Trip by site* | | | | | | | | | | |  | | | |  | | |  | | |  |  |  |
| Coral Gar. |  | | | | |  | |  | | |  | | | |  | | |  | | |  |  |  |
| Mar – Apr/May | -0.16 | | | | | 79 | | 0.99 | | | -1.46 | | | | 79 | | | 0.31 | | |  |  |  |
| Mar – Oct | 0.66 | | | | | 79 | | 0.79 | | | -0.58 | | | | 79 | | | 0.83 | | |  |  |  |
| Apr/May – Oct | 0.80 | | | | | 79 | | 0.70 | | | 0.89 | | | | 79 | | | 0.64 | | |  |  |  |
| North Bay |  | | | | |  | |  | | |  | | | |  | | |  | | |  |  |  |
| Mar – Apr/May | -3.27 | | | | | 79 | | **0.005** | | | -1.94 | | | | 79 | | | 0.13 | | |  |  |  |
| Mar – Oct | 1.89 | | | | | 79 | | 0.15 | | | 1.65 | | | | 79 | | | 0.23 | | |  |  |  |
| Apr/May – Oct | 5.31 | | | | | 79 | | **<0.0001** | | | 3.69 | | | | 79 | | | **0.001** | | |  |  |  |
| Sylph’s |  | | | | |  | |  | | |  | | | |  | | |  | | |  |  |  |
| Mar – Apr/May | 0.67 | | | | | 79 | | 0.78 | | | 1.18 | | | | 79 | | | 0.47 | | |  |  |  |
| Mar – Oct | 4.25 | | | | | 79 | | **0.0002** | | | 3.97 | | | | 79 | | | **0.0005** | | |  |  |  |
| Apr/May – Oct | 3.58 | | | | | 79 | | **0.0017** | | | 2.78 | | | | 79 | | | **0.02** | | |  |  |  |
| *Site by Trip* | | | | | | | | | | |  | | | |  | | |  | | |  |  |  |
| March |  | | | | |  | |  | | |  | | | |  | | |  | | |  |  |  |
| CG-NB | -0.47 | | | | | 79 | | 0.88 | | | -0.45 | | | | 79 | | | 0.89 | | |  |  |  |
| CG-Syl | -2.49 | | | | | 79 | | 0.39 | | | -3.69 | | | | 79 | | | **0.001** | | |  |  |  |
| NB-Syl | -1.95 | | | | | 79 | | 0.12 | | | -3.14 | | | | 79 | | | **0.007** | | |  |  |  |
| April/May |  | | | | |  | |  | | |  | | | |  | | |  | | |  |  |  |
| CG-NB | -3.58 | | | | | 79 | | **0.002** | | | -0.93 | | | | 79 | | | 0.62 | | |  |  |  |
| CG-Syl | -1.61 | | | | | 79 | | 0.25 | | | -0.98 | | | | 79 | | | 0.59 | | |  |  |  |
| NB-Syl | 2.02 | | | | | 79 | | 0.11 | | | -0.05 | | | | 79 | | | 0.99 | | |  |  |  |
| October |  | | | | |  | |  | | |  | | | |  | | |  | | |  |  |  |
| CG-NB | 0.81 | | | | | 79 | | 0.70 | | | 1.81 | | | | 79 | | | 0.17 | | |  |  |  |
| CG-Syl | 1.11 | | | | | 79 | | 0.51 | | | 0.85 | | | | 79 | | | 0.67 | | |  |  |  |
| NB-Syl | 0.299 | | | | | 79 | | 0.95 | | | -0.96 | | | | 79 | | | 0.60 | | |  |  |  |

**Table S6.** Table of results for *Cladiella* sp. 2 comparing Symbiodiniaceae per µg protein, chlorophyll a and chlorophyll c_2_ per µg protein, and chlorophyll a and chlorophyll c_2_ per Symbiodiniaceae cell. Non-significant interactions and factors not involved in interactions were removed from models. If models contained significant interactions pairwise comparisons were not conducted on single factors. Significant values are indicated in bold. Distributions used in GLMM models were as follows: Symbiodiniaceae – Poisson, all other factors – Gaussian. Significant results are highlighted in bold.

| Predictor | Symbiodiniaceae per µg protein | | | | | | | | Log(chlorophyll a per µg protein) | | | | | | | | | | Log(chlorophyll c per µg protein) | | | |
| --- | --- | --- | --- | --- | --- | --- | --- | --- | --- | --- | --- | --- | --- | --- | --- | --- | --- | --- | --- | --- | --- | --- |
| ANOVA **of GLMM** | | | | | | | | |  | | | | | | | | | |  | | | |
|  | χ^2^ | Df | | | | p-value | | | χ^2^ | | | | DF | | | p-value | | | χ^2^ | | DF | p-value |
| Monitoring interval | 4.04 | 2 | | | | 0.13 | | | 0.80 | | | | 2 | | | 0.67 | | | 21.01 | | 2 | **2.74E-05** |
| Site | 12.21 | 2 | | | | **0.002** | | | 3.44 | | | | 2 | | | 0.18 | | | 18.26 | | 2 | **0.0001** |
| Monitoring interval*Site | 3.2 | 4 | | | | 0.53 | | | 1.76 | | | | 4 | | | 0.78 | | | 6.63 | | 4 | 0.16 |
| **Pairwise comparisons** | | | | | | | | |  | | | |  | | |  | | |  | |  |  |
|  | t ratio | df | | | | p-value | | | t ratio | | | | df | | | p-value | | | t ratio | | df | p-value |
| *Site only* | | | | | | | | |  | | | |  | | |  | | |  | |  |  |
| CG-NB | -2.36 | 524 | | | | **0.049** | | | -1.56 | | | | 81 | | | 0.27 | | | -2.07 | | 80.9 | 0.10 |
| CG-Syl | -3.36 | 524 | | | | **0.002** | | | -1.65 | | | | 81 | | | 0.23 | | | -4.27 | | 81.1 | **0.0002** |
| NB-Syl | -1.03 | 524 | | | | 0.56 | | | -0.10 | | | | 81 | | | 0.99 | | | -2.20 | | 80.9 | 0.08 |
| *Monitoring interval only* | | | | | | | | |  | | | |  | | |  | | |  | |  |  |
| March-April/May | 0.42 | 524 | | | | 0.91 | | | 0.81 | | | | 81 | | | 0.69 | | | -0.98 | | 80.9 | 0.59 |
| March-October | 1.89 | 524 | | | | 0.14 | | | 0.74 | | | | 81 | | | 0.74 | | | 3.39 | | 80.9 | **0.003** |
| April/May-October | 1.48 | 524 | | | | 0.30 | | | -0.07 | | | | 81 | | | 0.99 | | | 4.36 | | 81.1 | **0.0001** |
| *Monitoring interval by site* | | | | | | | | |  | | | |  | | |  | | |  | |  |  |
| Coral Gar. |  |  | | | |  | | |  | | | |  | | |  | | |  | |  |  |
| March-April/May | 1.55 | 524 | | | | 0.27 | | | 1.07 | | | | 81 | | | 0.54 | | | -0.09 | | 80.7 | 0.99 |
| March-October | 1.73 | 524 | | | | 0.19 | | | 0.53 | | | | 81 | | | 0.86 | | | 0.81 | | 81.4 | 0.70 |
| April/May-October | 0.17 | 524 | | | | 0.98 | | | -0.55 | | | | 81 | | | 0.85 | | | 0.90 | | 81.4 | 0.64 |
| North Bay |  |  | | | |  | | |  | | | |  | | |  | | |  | |  |  |
| March-April/May | -0.35 | 524 | | | | 0.94 | | | -0.55 | | | | 81 | | | 0.85 | | | -1.88 | | 80.7 | 0.15 |
| March-October | 1.29 | 524 | | | | 0.40 | | | 0.23 | | | | 81 | | | 0.97 | | | 1.76 | | 80.7 | 0.19 |
| April/May-October | 1.65 | 524 | | | | 0.23 | | | 0.78 | | | | 81 | | | 0.72 | | | 3.64 | | 80.7 | **0.001** |
| Sylph’s |  |  | | | |  | | |  | | | |  | | |  | | |  | |  |  |
| March-April/May | -0.47 | 524 | | | | 0.88 | | | 0.88 | | | | 81 | | | 0.66 | | | 0.28 | | 81.4 | 0.96 |
| March-October | 0.26 | 524 | | | | 0.96 | | | 0.52 | | | | 81 | | | 0.86 | | | 3.31 | | 80.7 | **0.004** |
| April/May-October | 0.76 | 524 | | | | 0.73 | | | -0.36 | | | | 81 | | | 0.93 | | | 3.02 | | 81.4 | **0.009** |
| *Site by Monitoring interval* | | | | | | | | |  | | | |  | | |  | | |  | |  |  |
| 1 |  |  | | | |  | | |  | | | |  | | |  | | |  | |  |  |
| CG-NB | -0.57 | 524 | | | | 0.84 | | | -0.26 | | | | 81 | | | 0.96 | | | -0.92 | | 80.7 | 0.63 |
| CG-Syl | -0.76 | 524 | | | | 0.73 | | | -0.89 | | | | 81 | | | 0.65 | | | -3.42 | | 80.7 | **0.003** |
| NB-Syl | -0.20 | 524 | | | | 0.98 | | | -0.63 | | | | 81 | | | 0.80 | | | -2.50 | | 80.7 | 0.04 |
| 2 |  |  | | | |  | | |  | | | |  | | |  | | |  | |  |  |
| CG-NB | -2.49 | 524 | | | | **0.03** | | | -1.88 | | | | 81 | | | 0.15 | | | -2.71 | | 80.7 | **0.02** |
| CG-Syl | -2.85 | 524 | | | | **0.01** | | | -1.08 | | | | 81 | | | 0.53 | | | -3.05 | | 81.4 | **0.009** |
| NB-Syl | -0.35 | 524 | | | | 0.94 | | | 0.80 | | | | 81 | | | 0.71 | | | -0.35 | | 81.4 | 0.94 |
| 3 |  |  | | | |  | | |  | | | |  | | |  | | |  | |  |  |
| CG-NB | -1.03 | 524 | | | | 0.56 | | | -0.56 | | | | 81 | | | 0.84 | | | 0.031 | | 81.4 | 0.99 |
| CG-Syl | -2.27 | 524 | | | | 0.6 | | | -0.89 | | | | 81 | | | 0.66 | | | -0.92 | | 81.4 | 0.63 |
| NB-Syl | -0.24 | 524 | | | | 0.43 | | | -0.34 | | | | 81 | | | 0.94 | | | -0.96 | | 80.7 | 0.61 |
| Predictor | chlorophyll a per Symbiodiniaceae cell | | | | | | | | | chlorophyll c_2_ per Symbiodiniaceae cell | | | | | | | | | | |  |  |
|  | Sum sq | Mean sq | Df | | F value | | | P value | | Sum sq | | Mean sq | | | Df | | F value | | | P value |  |  |
| Monitoring interval | 3.28e-11 | 1.64e-11 | 2 | | 13.61 | | | **<0.0001** | | 1.23e-12 | | 6.15e-13 | | | 2 | | 6.99 | | | **0.002** |  |  |
| Site | 1.59e-11 | 7.96e-12 | 2 | | 6.60 | | | **0.002** | | 0.81e-12 | | 9.04e-13 | | | 2 | | 10.28 | | | **0.0001** |  |  |
| Monitoring interval*Site | 2.35e-11 | 5.87e-12 | 4 | | 4.87 | | | **0.001** | | 0.63e-12 | | 4.07e-13 | | | 4 | | 4.62 | | | **0.002** |  |  |
| Residuals | 19.65e-10 | 1.21e-12 | 80 | |  | | |  | | 7.12e-12 | | 8.79e-13 | | | 81 | |  | | |  |  |  |
| **Pairwise comparisons** | | | | | | | | | | |  | | | | | | | | | |  |  |
|  | t ratio | df | | p-value | | | | | | t ratio | | | | df | | | | p-value | | |  |  |
| *Site only* | | | | | | |  | | | | | | |  | | | |  | | |  |  |
| CG-NB | -- | -- | | -- | | | | | | -- | | | | -- | | | | -- | | |  |  |
| CG-Syl | -- | -- | | -- | | | | | | -- | | | | -- | | | | -- | | |  |  |
| NB-Syl | -- | -- | | -- | | | | | | -- | | | | -- | | | | -- | | |  |  |
| *Monitoring interval only* | | | | | | |  | | | | | | |  | | | |  | | |  |  |
| March-April/May | -- | -- | | -- | | | | | | -- | | | | -- | | | | -- | | |  |  |
| March-October | -- | -- | | -- | | | | | | -- | | | | -- | | | | -- | | |  |  |
| April/May-October | -- | -- | | -- | | | | | | -- | | | | -- | | | | -- | | |  |  |
| *Monitoring interval by site* | | | | | | |  | | | | | | |  | | | |  | | |  |  |
| Coral Gar. |  |  | |  | | | | | |  | | | |  | | | |  | | |  |  |
| March-April/May | -0.59 | 80 | | 0.86 | | | | | | -1.46 | | | | 81 | | | | 0.31 | | |  |  |
| March-October | -1.77 | 80 | | 0.19 | | | | | | -0.58 | | | | 81 | | | | 0.83 | | |  |  |
| April/May-October | -1.24 | 80 | | 0.43 | | | | | | 0.89 | | | | 81 | | | | 0.64 | | |  |  |
| North Bay |  |  | |  | | | | | |  | | | |  | | | |  | | |  |  |
| March-April/May | 4.52 | 80 | | **0.0001** | | | | | | -1.94 | | | | 81 | | | | 0.13 | | |  |  |
| March-October | -1.49 | 80 | | 0.30 | | | | | | 1.65 | | | | 81 | | | | 0.23 | | |  |  |
| April/May-October | -5.97 | 80 | | **<0.0001** | | | | | | 3.69 | | | | 81 | | | | **0.001** | | |  |  |
| Sylph’s |  |  | |  | | | | | |  | | | |  | | | |  | | |  |  |
| March-April/May | 2.24 | 80 | | 0.07 | | | | | | 1.19 | | | | 81 | | | | 0.47 | | |  |  |
| March-October | 0.37 | 80 | | 0.93 | | | | | | 3.97 | | | | 81 | | | | **0.0005** | | |  |  |
| April/May-October | -1.67 | 80 | | 0.15 | | | | | | 2.78 | | | | 81 | | | | **0.02** | | |  |  |
| *Site by monitoring interval* | | | | | | |  | | | | | | |  | | | |  | | |  |  |
| March |  |  | |  | | | | | |  | | | |  | | | |  | | |  |  |
| CG-NB | 0.34 | 80 | | 0.94 | | | | | | -0.71 | | | | 81 | | | | 0.76 | | |  |  |
| CG-Syl | -0.32 | 80 | | 0.95 | | | | | | -4.81 | | | | 81 | | | | **<0.0001** | | |  |  |
| NB-Syl | -0.65 | 80 | | 0.79 | | | | | | -4.11 | | | | 81 | | | | **0.0003** | | |  |  |
| April/May |  |  | |  | | | | | |  | | | |  | | | |  | | |  |  |
| CG-NB | 5036 | 80 | | **<0.0001** | | | | | | 1.75 | | | | 81 | | | | 0.19 | | |  |  |
| CG-Syl | 2.45 | 80 | | **0.04** | | | | | | -1.58 | | | | 81 | | | | 0.26 | | |  |  |
| NB-Syl | -2.98 | 80 | | **0.01** | | | | | | -3.33 | | | | 81 | | | | **0.004** | | |  |  |
| October |  |  | |  | | | | | |  | | | |  | | | |  | | |  |  |
| CG-NB | 0.61 | 80 | | 0.81 | | | | | | 0.87 | | | | 81 | | | | 0.65 | | |  |  |
| CG-Syl | 1.82 | 80 | | 0.17 | | | | | | 0.76 | | | | 81 | | | | 0.73 | | |  |  |
| NB-Syl | 1.21 | 80 | | 0.45 | | | | | | 0.12 | | | | 81 | | | | 0.99 | | |  |  |

**Table S7.** Statistical results of photosynthetic yield and protein per wet weight in *Xenia* cf *crassa.* All tests were performed using a Gaussian distribution.

|  | Photosynthetic yield | | | Protein per wet weight | | | | | |
| --- | --- | --- | --- | --- | --- | --- | --- | --- | --- |
| ANOVA **of lmer (yield) or** ANOVA **(protein)** | | | | | | | | | |
|  | χ^2^ | Df | p-value | Sum sq | Mean sq | | Df | F value | P value |
| Trip | 2.17 | 2 | 0.34 | 3.6e4 | 1.8e4 | | 2 | 1.47 | 0.24 |
| Site | 3.30 | 2 | 0/19 | 4.9e5 | 2.5e5 | | 2 | 20.34 | **7.51e-08** |
| Trip*Site | 7.98 | 3 | **0.046** | 1.9e4 | 4663 | | 4 | 0.39 | 0.82 |
| Residuals | -- |  |  | 9.6e6 | 1.2e4 | | 79 |  |  |
|  |  |  |  |  | |  | |  | |
|  | T ratio | Df | p-value | T ratio | | Df | | p-value | |
| *Site only* |  | | |  | |  | |  | |
| CG-NB | -- | -- | -- | -1.37 | | 79 | | 0.37 | |
| CG-Syl | -- | -- | -- | -1.18 | | 79 | | 0.47 | |
| NB-Syl | -- | -- | -- | 0.19 | | 79 | | 0.98 | |
| *Trip only* |  | | |  | |  | |  | |
| 1-2 | -- | -- | -- | -6.40 | | 79 | | **<0.0001** | |
| 1-3 | -- | -- | -- | -3.46 | | 79 | | **0.002** | |
| 2-3 | -- | -- | -- | 2.99 | | 79 | | **0.0101** | |
| *Trip by site* |  | | |  | |  | |  | |
| Coral Gar. |  |  |  |  | |  | |  | |
| 1-2 | 0.22 | 70.7 | 0.97 | -3.85 | | 79 | | **0.0007** | |
| 1-3 | -1.03 | 70.7 | 0.56 | -1.27 | | 79 | | 0.42 | |
| 2-3 | -1.22 | 70.7 | 0.44 | 2.61 | | 79 | | **0.03** | |
| North Bay |  |  |  |  | |  | |  | |
| 1-2 | -- | -- | -- | -3.82 | | 79 | | **0.0008** | |
| 1-3 | -- | -- | -- | -2.47 | | 79 | | **0.04** | |
| 2-3 | 1.13 | 70.7 | 0.50 | 1.39 | | 79 | | 0.35 | |
| Sylph’s |  |  |  |  | |  | |  | |
| 1-2 | 1.26 | 71.8 | 0.42 | -3.42 | | 79 | | **0.003** | |
| 1-3 | 2.67 | 71.8 | **0.03** | -2.24 | | 79 | | 0.07 | |
| 2-3 | 1.41 | 70.7 | 0.34 | 1.18 | | 79 | | 0.47 | |
| *Site by Trip* |  | | |  | |  | |  | |
| 1 |  |  |  |  | |  | |  | |
| CG-NB | -- | -- | -- | -19.05 | | 79 | | 0.92 | |
| CG-Syl | -0.54 | 71.8 | 0.85 | -26.72 | | 79 | | 0.85 | |
| NB-Syl | -- | -- | -- | -7.67 | | 79 | | 0.99 | |
| 2 |  |  |  |  | |  | |  | |
| CG-NB | -0.36 | 70.7 | 0.93 | -17.14 | | 79 | | 0.94 | |
| CG-Syl | 0.48 | 70.7 | 0.88 | -0.38 | | 79 | | 0.99 | |
| NB-Syl | 0.86 | 70.7 | 0.67 | 17.26 | | 79 | | 0.93 | |
| 3 |  |  |  |  | |  | |  | |
| CG-NB | 2.02 | 70.7 | 0.12 | -81.13 | | 79 | | 0.23 | |
| CG-Syl | 3.16 | 70.7 | **0.007** | -74.41 | | 79 | | 0.29 | |
| NB-Syl | 1.15 | 70.7 | 0.49 | 6.73 | | 79 | | 0.99 | |

**Table S7.** Statistical results of photosynthetic yield and protein per wet weight in *Cladiella* sp. 2*.* All tests were performed using a Gaussian distribution. Significant results are highlighted in bold.

|  | Photosynthetic yield | | | Protein per wet weight | | | | |
| --- | --- | --- | --- | --- | --- | --- | --- | --- |
| ANOVA **of lmer (yield) or** ANOVA **(protein)** | | | |  | | | | |
|  | χ^2^ | df | P-value | Sum sq | Mean sq | Df | F value | P value |
| Site | 1.85 | 2 | 0.39 | 5.5e5 | 2.8e5 | 2 | 0.141 | 0.87 |
| Trip | 21.45 | 2 | **2.19e-05** | 1.8e7 | 8.9e6 | 2 | 33.2 | **3.43e-11** |
| Trip*Site | 61.36 | 3 | **3.1e-13** | 1.5e6 | 3.7e5 | 4 | 2.13 | 0.09 |
| Residuals |  |  |  | 2.1e7 | 2.7e5 | 79 |  |  |
|  |  |  |  |  |  |  |  |  |
|  | t-ratio | Df | p-value | t ratio | df | p-value | | |
| *Site only* |  | | | | | | | |
| CG-NB | -- | -- | -- | -0.82 | 79 | 0.69 | | |
| CG-Syl | -- | -- | -- | -1.06 | 79 | 0.54 | | |
| NB-Syl | -- | -- | -- | -0.25 | 79 | 0.97 | | |
| *Trip only* |  | | | | | | | |
| 1-2 | -- | -- | -- | -2.48 | 79 | **0.04** | | |
| 1-3 | -- | -- | -- | -8.00 | 79 | **<0.0001** | | |
| 2-3 | -- | -- | -- | -5.59 | 79 | **<0.0001** | | |
| *Trip by site* |  | | | | | | | |
| Coral Gar. |  |  |  |  |  |  | | |
| 1-2 | 1.04 | 72.1 | 0.55 | -1.65 | 79 | 0.23 | | |
| 1-3 | 3.69 | 72.1 | **0.001** | -6.01 | 79 | **<0.0001** | | |
| 2-3 | 2.66 | 71.5 | **0.03** | -4.40 | 79 | **0.0001** | | |
| North Bay |  |  |  |  |  |  | | |
| 1-2 | -- | -- | -- | -0.56 | 79 | 0.84 | | |
| 1-3 | -- | -- | -- | -4.08 | 79 | **0.0003** | | |
| 2-3 | -5.60 | 72.1 | **<0.0001** | -3.52 | 79 | **0.002** | | |
| Sylph’s |  |  |  |  |  |  | | |
| 1-2 | -2.90 | 72.1 | **0.01** | -2.07 | 79 | 0.10 | | |
| 1-3 | 1.21 | 72.1 | 0.50 | -3.76 | 79 | **0.001** | | |
| 2-3 | 4.03 | 71.5 | **0.0004** | -1.72 | 79 | 0.20 | | |
| *Site by Trip* |  | | | | | | | |
| 1 |  |  |  |  |  |  | | |
| CG-NB | -- | -- | -- | -1.53 | 79 | 0.28 | | |
| CG-Syl | 3.22 | 72.8 | **0.006** | -1.20 | 79 | 0.46 | | |
| NB-Syl | -- | -- | -- | 0.29 | 79 | 0.95 | | |
| 2 |  |  |  |  |  |  | | |
| CG-NB | 1.52 | 72.1 | 0.29 | -0.45 | 79 | 0.90 | | |
| CG-Syl | -0.72 | 71.5 | 0.75 | -1.71 | 79 | 0.21 | | |
| NB-Syl | -2.24 | 72.1 | 0.07 | -1.27 | 79 | 0.42 | | |
| 3 |  |  |  |  |  |  | | |
| CG-NB | -6.75 | 71.5 | **<0.0001** | 0.52 | 79 | 0.85 | | |
| CG-Syl | 0.66 | 71.5 | 0.79 | 1.05 | 79 | 0.55 | | |
| NB-Syl | 7.41 | 71.5 | **0.0004** | 0.53 | 79 | 0.86 | | |

**Figures**

**
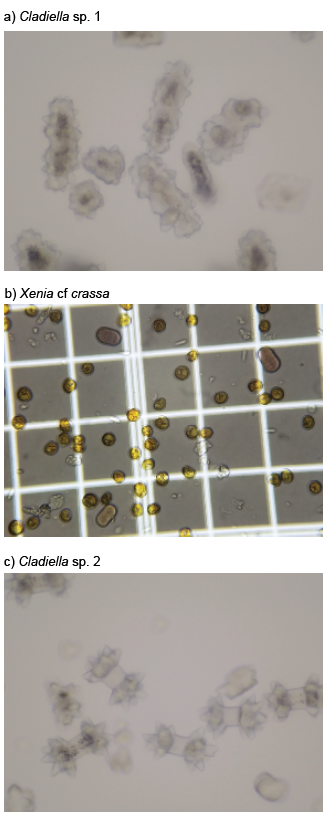
**

**Figure S1: Sclerites of Lord Howe Island Octocorals.** a) *Cladiella* sp. 1 sclerites, b) *Xenia* cf *crassa* sclerites and Symbiodiniaceae. Sclerites are oval-shaped and brownish while Symbiodiniaceae are round and green, c) *Cladiella* sp. 2 sclerites.


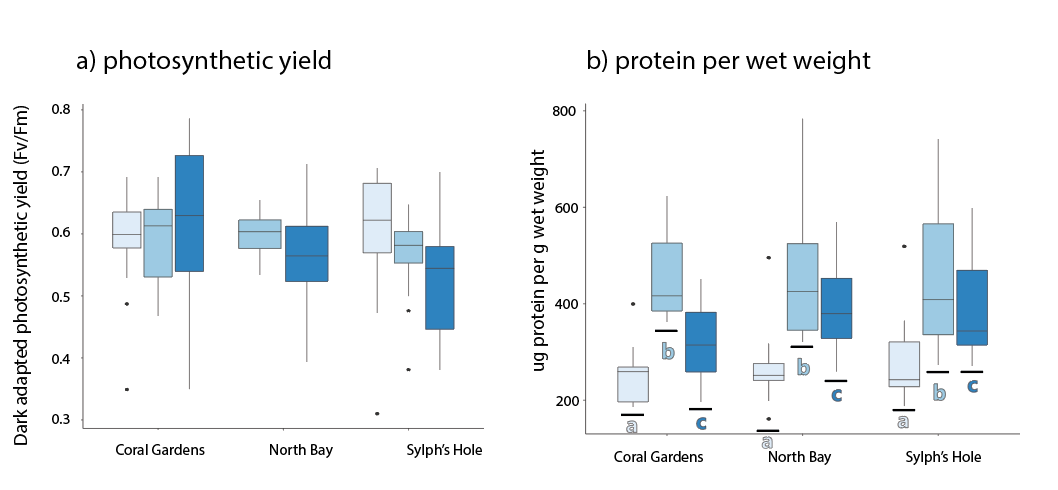


**Figure S2: *Xenia* cf *crassa****,* a) photosynthetic yield and b) protein per wet weight.


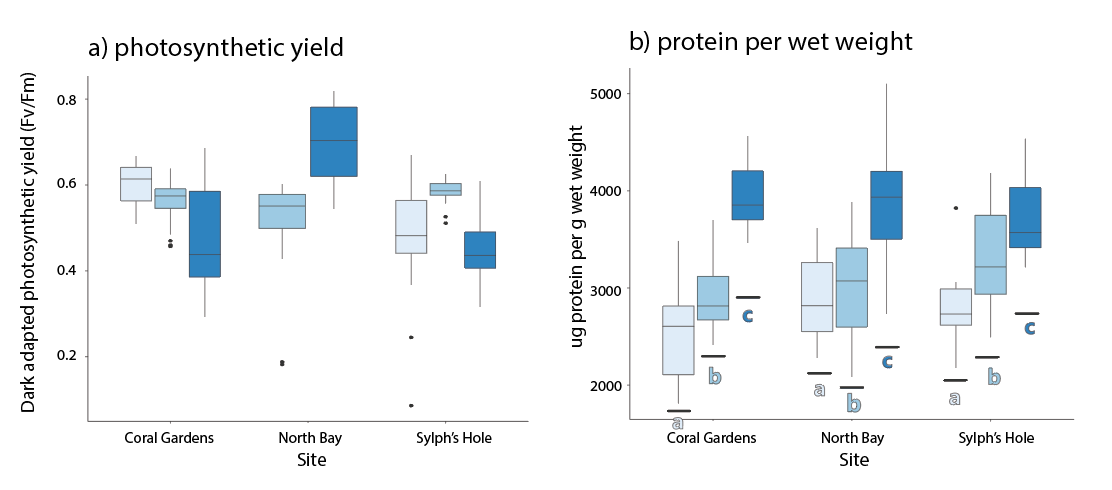


**Figure S3: *Cladiella* sp. 2**, a) photosynthetic yield and b) protein per wet weight.

**Video S1: Cladiella peeling from the benthos.** Video available at <https://youtu.be/rjp7XdKMcLc>. Video taken by Tess Moriarty.
